# Self-Assembly of the RZZ Complex into Filaments Drives Kinetochore Expansion in the Absence of Microtubule Attachment

**DOI:** 10.1016/j.cub.2018.08.056

**Published:** 2018-11-05

**Authors:** Cláudia Pereira, Rita M. Reis, José B. Gama, Ricardo Celestino, Dhanya K. Cheerambathur, Ana X. Carvalho, Reto Gassmann

**Affiliations:** 1 Instituto de Biologia Molecular e Celular (IBMC), Universidade do Porto, 4200-135 Porto, Portugal; 2 Instituto de Investigação e Inovação em Saúde (i3S), Universidade do Porto, 4200-135 Porto, Portugal; 3 Ludwig Institute for Cancer Research, Department of Cellular and Molecular Medicine, University of California San Diego, La Jolla, CA 92093, USA

**Keywords:** mitosis, kinetochore, RZZ complex, spindly, dynein, fibrous corona, spindle assembly checkpoint

## Abstract

The kinetochore is a dynamic multi-protein assembly that forms on each sister chromatid and interacts with microtubules of the mitotic spindle to drive chromosome segregation. In animals, kinetochores without attached microtubules expand their outermost layer into crescent and ring shapes to promote microtubule capture and spindle assembly checkpoint (SAC) signaling. Kinetochore expansion is an example of protein co-polymerization, but the mechanism is not understood. Here, we present evidence that kinetochore expansion is driven by oligomerization of the Rod-Zw10-Zwilch (RZZ) complex, an outer kinetochore component that recruits the motor dynein and the SAC proteins Mad1-Mad2. Depletion of ROD in human cells suppresses kinetochore expansion, as does depletion of Spindly, the adaptor that connects RZZ to dynein, although dynein itself is dispensable. Expansion is also suppressed by mutating ZWILCH residues implicated in Spindly binding. Conversely, supplying cells with excess ROD facilitates kinetochore expansion under otherwise prohibitive conditions. Using the *C. elegans* early embryo, we demonstrate that ROD-1 has a concentration-dependent propensity for oligomerizing into micrometer-scale filaments, and we identify the ROD-1 β-propeller as a key regulator of self-assembly. Finally, we show that a minimal ROD-1-Zw10 complex efficiently oligomerizes into filaments *in vitro*. Our results suggest that RZZ’s capacity for oligomerization is harnessed by kinetochores to assemble the expanded outermost domain, in which RZZ filaments serve as recruitment platforms for SAC components and microtubule-binding proteins. Thus, we propose that reversible RZZ self-assembly into filaments underlies the adaptive change in kinetochore size that contributes to chromosome segregation fidelity.

## Highlights

• The Rod-Zw10-Zwilch complex is required for kinetochore expansion in human cells
• Over-expression of the RZZ subunit Rod promotes expansion of unattached kinetochores
• *C. elegans* RZZ is capable of reversible self-assembly into micrometer-scale filaments
• A minimal *C. elegans* Rod-Zw10 complex is sufficient for self-assembly *in vitro*

## Teaser

Unattached kinetochores are known to expand their outermost layer to accelerate spindle assembly. Pereira, Reis, et al. present evidence suggesting that self-assembly of the Rod-Zw10-Zwilch complex into filaments, driven by the Rod subunit that is structurally related to membrane coat proteins, underlies the adaptive change in kinetochore size.

## Introduction

Equal segregation of chromosomes to daughter cells during cell division requires interactions between spindle microtubules and the kinetochore, a complex multi-protein assembly that forms at the centromeric locus of each sister chromatid. In animal cells, kinetochores only gain access to microtubules once the nuclear envelope breaks down. For timely bi-orientation of sister chromatids on the mitotic spindle, kinetochores must be efficient at capturing microtubules and converting initial lateral microtubule contacts to the stable end-on attachments that drive chromosome segregation [1]. As a safeguard, the spindle assembly checkpoint (SAC) generates an inhibitory "wait anaphase" signal at kinetochores that have not yet established stable attachments to microtubules, thereby ensuring that the cell proceeds with anaphase and mitotic exit only once all sister kinetochores have bi-oriented [2]. Elucidating the molecular mechanisms that promote microtubule capture and SAC signaling at kinetochores remains an important challenge.

Kinetochores assemble in a largely hierarchical manner starting from CENP-A-containing nucleosomes and the associated protein CENP-C, which is part of the 16-subunit constitutive centromere-associated network (CCAN) (also referred to as the inner kinetochore) [3]. The CCAN then directs the assembly of the microtubule interface (or outer kinetochore), consisting of the ten-subunit Knl1 complex, Mis12 complex, Ndc80 complex (KMN) network. The KMN network mediates end-on microtubule attachment through the Ndc80 complex and provides a platform for generation of the mitotic checkpoint complex (MCC), consisting of BubR1 (also known as Mad3), Bub3, Mad2, and Cdc20, which prevents mitotic exit by inhibiting the E3 ubiquitin ligase anaphase-promoting complex/cyclosome (APC/C) [4]. Knl1 acts as the scaffold for the recruitment of Bub3, BubR1, and the kinase Bub1 [5], which is thought to promote MCC formation by directly binding to a Mad1-Mad2 template that catalyzes MCC assembly [6, 7, 8, 9, 10]. Knl1 and Bub1 are also implicated in the recruitment of the three-subunit Rod-Zw10-Zwilch (RZZ) complex [11, 12, 13, 14, 15], which is one of several components that localize peripherally to the KMN network and include the microtubule-associated protein (MAP) CENP-F and the microtubule motors dynein and CENP-E. RZZ recruits dynein and its co-factor dynactin through a direct interaction with the dynein adaptor Spindly [15, 16]. RZZ is also required for Mad1-Mad2 recruitment, and recent work suggests that RZZ’s contribution to SAC signaling may be distinct from that of Bub1 [13, 17].

Kinetochores have long been known to respond to the absence of microtubules by expanding into ring and crescent shapes that encircle the centromere [18, 19]. Kinetochore expansion occurs through addition of new material rather than re-distribution of existing protein and is characterized by the formation of an outermost domain, called the fibrous corona based on its appearance in electron microscopy [18, 20]. Kinetochore expansion is particularly prominent when mitosis with unattached kinetochores is prolonged by treatment with microtubule depolymerizing drugs [20, 21], but kinetochores also expand in unperturbed cells in early prometaphase, when kinetochores are devoid of microtubules [22]. Kinetochore expansion is proposed to make two critical contributions to the fidelity of chromosome segregation: it increases the amount of kinetochore-localized SAC proteins, including Mad1 and Mad2, implying that more MCC can be generated per unattached kinetochore, and it distributes MAPs, such as CENP-F, CENP-E, and dynein, over a larger surface, which facilitates lateral microtubule capture [19, 21, 22, 23, 24]. Consequently, kinetochore expansion is predicted to both expedite the formation of end-coupled kinetochore-microtubule attachments and amplify the checkpoint signal [21, 22], which may be particularly relevant when anaphase has to be delayed in the presence of just a few or even a single unattached kinetochore [25].

Mechanistically, kinetochore expansion remains poorly understood but is thought to be an example of multi-protein co-polymerization [21]. Recent work using super-resolution light microscopy in *Xenopus* egg extracts identified an expandable kinetochore module consisting of micrometer-long fibers that grow out from centromeric chromatin along chromosome arms. Fibrous extensions emanating from mitotic chromosomes have also been observed in *C. elegans* embryos treated with nocodazole [26], and filaments containing kinetochore components surround chromosomes in the *C. elegans* meiosis I embryo [27, 28]. Intriguingly, recent analysis of reconstituted human RZZ by cryo-electron microscopy confirmed an earlier prediction that the Rod subunit is structurally related to membrane coat proteins such as Clathrin and subunits of the COPI and COPII complexes [16, 29]. The underlying common design, which consists of an N-terminal β-propeller domain and C-terminally located α-solenoid motifs, enables coat proteins to form higher order assemblies around vesicles that act as scaffolds to direct membrane traffic [30, 31].

Here, using cultured human cells, the *C. elegans* early embryo, and purified proteins, we demonstrate that RZZ is capable of oligomerizing into micrometer-scale filaments and present evidence that Rod is the critical subunit for self-assembly, as predicted by its architectural similarity with membrane coat proteins. Our results suggest that RZZ’s propensity for oligomerization is harnessed at kinetochores to drive the assembly of the expanded outer domain, in which RZZ filaments serve as platforms for the recruitment of SAC proteins and MAPs.

## Results

### Kinetochore Expansion Requires the RZZ Complex and SPDL1, but Not Dynein-Dynactin

**Figure 1:**
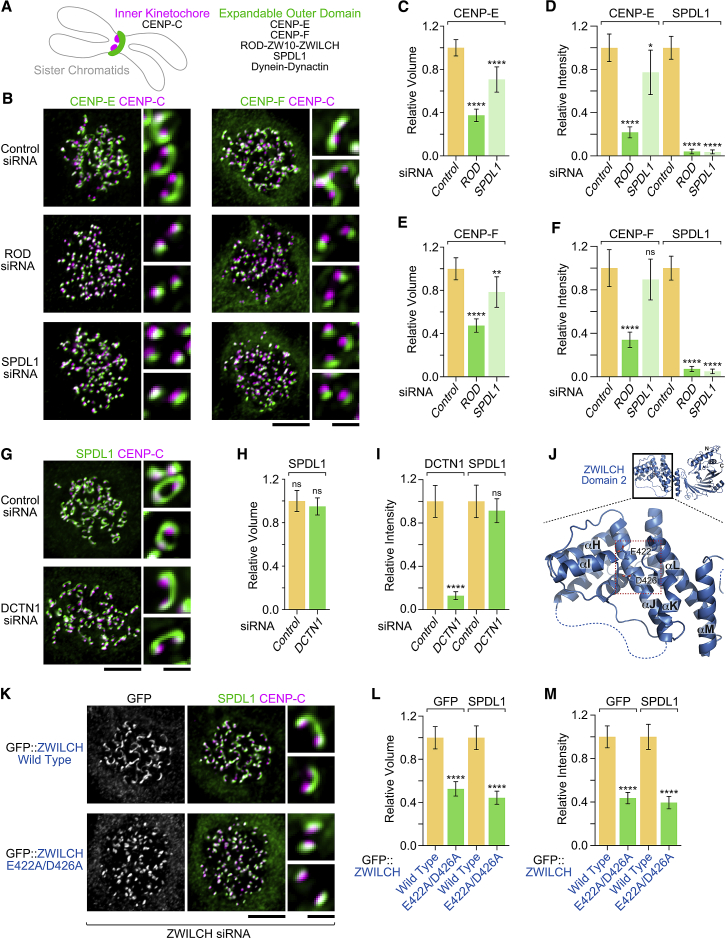
Kinetochore Expansion Requires the RZZ Complex and SPDL1 but Is Independent of Dynein-Dynactin (A) Cartoon showing the crescent shape characteristic of the expanded outer kinetochore, which encircles the compact inner kinetochore. Components analyzed in this figure are listed on the right. (B) Immunofluorescence images showing that kinetochore expansion in nocodazole is inhibited after RNAi-mediated depletion of ROD or SPDL1. Scale bars, 5 μm; blow-ups, 1 μm. (C–F) Quantification of relative kinetochore volume for CENP-E (C) and CENP-F (E) and kinetochore signal intensity for CENP-E (D) and CENP-F (F), based on fluorescence measurements in images as shown in (B). For each condition, the mean value per kinetochore was determined for individual cells. Final values are shown as the mean of mean (n = 20 cells), normalized to the control. Error bars denote the 95% confidence interval. Statistical significance was determined by one-way ANOVA followed by Bonferroni’s multiple comparison test. ^∗∗∗∗^p < 0.0001; ^∗∗^p < 0.01; ^∗^p < 0.05; not significant (ns), p > 0.05. (G) Immunofluorescence images showing that depletion of the dynactin subunit DCTN1 does not affect kinetochore expansion. Scale bars, 5 μm; blow-ups, 1 μm. (H and I) Quantification of relative kinetochore volume (H) and signal intensity (I), determined and plotted as described for (C)–(F). The t test was used to determine statistical significance. ^∗∗∗∗^p < 0.0001; ns, p > 0.05. (J) Cartoon model of ZWILCH (PDB: 3IF8) obtained with PyMOL. Dotted lines mark loop regions that were not visible in the crystal structure and were added to improve clarity. The red boxed region indicates the position of E422 and D426. (K) Immunofluorescence images showing that the ZWILCH mutant E422A/D426A does not support kinetochore expansion. Scale bars, 5 μm; blow-ups, 1 μm. (L and M) Quantification of relative kinetochore volume (L) and signal intensity (M), determined and plotted as described for (C)–(F). The t test was used to determine statistical significance. ^∗∗∗∗^p < 0.0001. See also Figure S1.

To examine the role of the kinetochore dynein module (RZZ-SPDL1-dynein-dynactin) in kinetochore expansion, we incubated HeLa cells with nocodazole to depolymerize microtubules and used immunostaining for the outer kinetochore proteins CENP-E and CENP-F to assess crescent formation (Figure 1A). In cells treated with control small interfering RNA (siRNA), CENP-E and CENP-F expanded into crescents that partially encircled the compact inner kinetochore, marked by CENP-C, as expected (Figure 1B). Depletion of the RZZ subunit ROD by RNAi, which eliminated SPDL1 localization to kinetochores, supported CENP-E and CENP-F recruitment, but kinetochores no longer expanded into crescents (Figures 1B and S1A). Measurements of kinetochore fluorescence confirmed that ROD depletion reduced both the volume occupied by CENP-E and CENP-F and their overall levels (Figures 1C–1F). Depletion of SPDL1 also reduced kinetochore expansion, albeit not to the same degree as depletion of ROD (Figures 1B–1F and S1B). By contrast, depletion of the dynactin subunit DCTN1, which prevents kinetochore recruitment of both dynactin and dynein, did not affect kinetochore expansion, as judged by immunostaining for SPDL1 (Figures 1G–1I and S1C). We conclude that kinetochore expansion requires RZZ and SPDL1 but is independent of dynein-dynactin.

### ZWILCH Residues Implicated in SPDL1 Binding Are Required for Kinetochore Expansion

Human SPDL1 directly interacts with the ROD β-propeller [16], and the β-propeller is essential for SPDL1 recruitment to kinetochores [15]. The atomic-resolution structure of the RZZ subunit ZWILCH, which forms a tight complex with the ROD β-propeller, features a conserved surface-exposed patch of unknown function in domain 2 [29] (Figure 1J). In *C. elegans*, two acidic residues in this patch are required for an interaction between ZWL-1^Zwilch^ and SPDL-1 [15]. We used the Flp-In system in HeLa cells and RNAi-based molecular replacement to assess the consequences of introducing analogous mutations (E422A/D426A) into human ZWILCH (Figure 1J). Expression of transgene-encoded GFP::ZWILCH, harboring silent mutations that confer RNAi resistance, supported recruitment of SPDL1 to kinetochores and facilitated kinetochore expansion in nocodazole (Figures 1K–1M and S1D). By contrast, GFP::ZWILCH (E422A/D426A), although supporting SPDL1 recruitment to kinetochores, inhibited kinetochore expansion to a similar degree as depletion of ROD (Figures 1K–1M and S1E). We conclude that kinetochore expansion requires two conserved acidic ZWILCH residues that, in *C. elegans* ZWL-1^Zwilch^, are implicated in an interaction with SPDL-1.

### The Expanded Outer Kinetochore Is a Distinct Multi-protein Domain that Can Be Dissociated from the KMN Network

**Figure 2:**
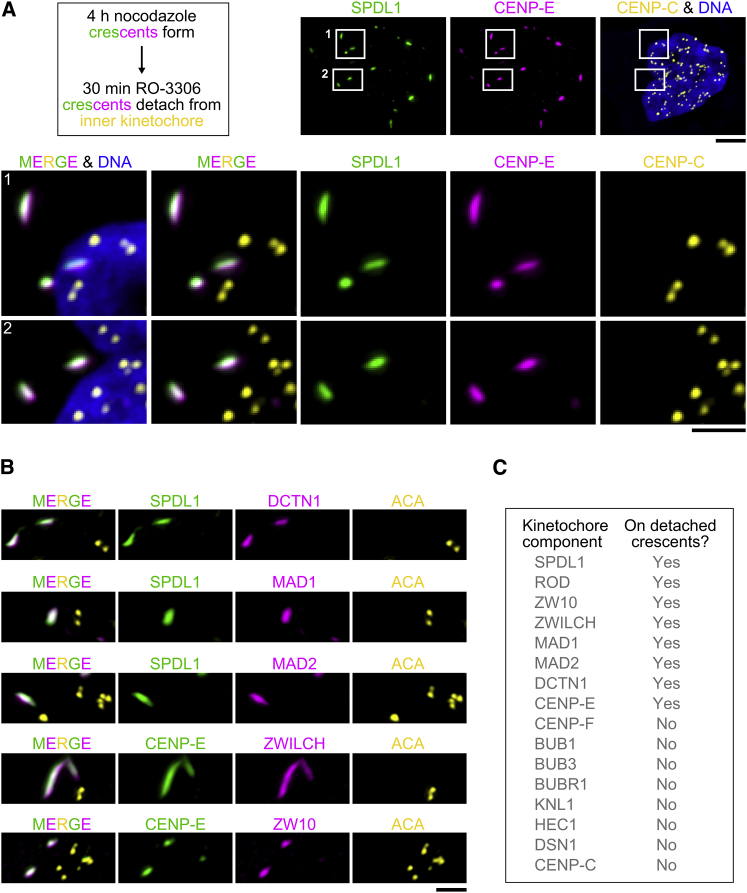
The Expanded Outer Kinetochore Is a Distinct Multi-protein Domain that Can Be Dissociated from the KMN Network (A) (Left) Summary of the experimental regime to detach the expanded kinetochore domain (crescents) from the centromere. (Right and bottom) Immunofluorescence images showing crescents, marked by CENP-E and SPDL1, that are fully detached from the inner kinetochore, marked by CENP-C. Boxed regions are shown separately at higher magnification. Scale bars, 5 μm; blow-ups, 2 μm. (B) Immunofluorescence images showing the composition of detached crescents. Scale bar, 2 μm. (C) Table summarizing the protein composition of detached crescents, based on immunofluorescence analysis. See also Figures S1 and S2.

We found that, following a 4-hr incubation in nocodazole, a 30-min treatment of mitotic cells with the CDK1 inhibitor RO-3306 resulted in complete detachment of the expanded outer domain from the centromere (Figures 2A, S1F, and S1G). Immunostaining showed that crescent-like assemblies in the cytoplasm, separated by several micrometers from the closest sister kinetochore pair, marked by CENP-C or human anti-centromere antibodies (ACA), contained ZW10, ZWILCH, SPDL1, the dynactin subunit DCTN1, CENP-E, MAD1, and MAD2 (Figures 2B and 2C). By contrast, we could not detect KNL1, BUB1, BUBR1, BUB3, CENP-F, HEC1, DSN1, or CENP-C on detached crescents (Figures 2C and S2). With the exception of CENP-C, these components were also missing from kinetochores after CDK1 inhibition, consistent with a previous study that used acute CDK1 inhibition to probe kinetochore assembly [32]. Thus, the expanded outer domain most likely detaches after RO-3306 treatment because it is no longer tethered to kinetochores by the KMN network. There were generally fewer detached crescents than kinetochores (Figure S1F), perhaps indicating that some crescents fuse with each other after being shed from the kinetochore. Detached crescents were never observed after depletion of ROD or SPDL1, consistent with their role in kinetochore expansion. We conclude that kinetochore expansion involves the assembly of a distinct multi-protein domain with defined composition that is separable from the inner kinetochore and the KMN network. Moreover, the robust presence of MAD1-MAD2 on detached kinetochore crescents in the absence of detectable KNL1 or BUB1 implies that RZZ has a direct role in the retention of MAD1-MAD2 at kinetochores, consistent with previous work [14, 17].

### Expression of Exogenous ROD Promotes Kinetochore Expansion

**Figure 3:**
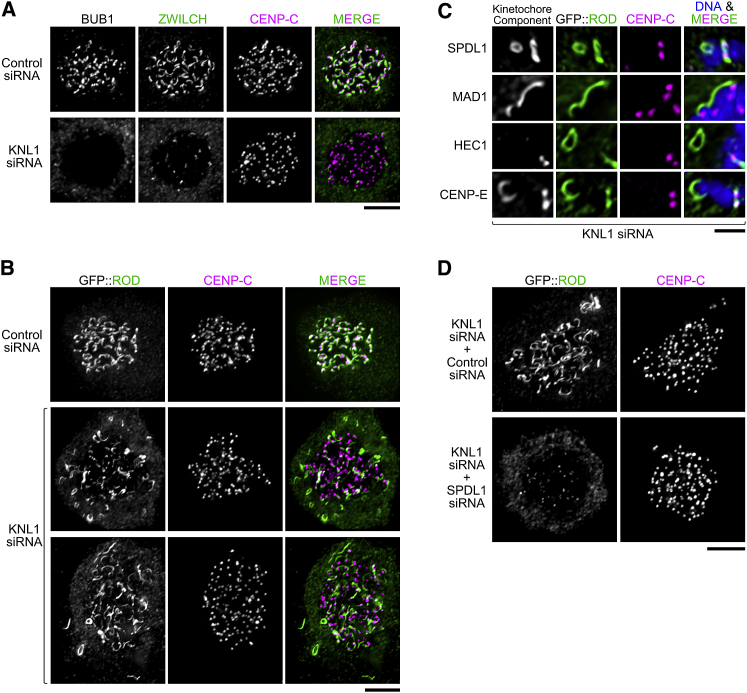
Expression of Exogenous GFP::ROD Promotes Kinetochore Expansion (A) Immunofluorescence images showing that KNL1 depletion reduces RZZ levels at kinetochores and prevents kinetochore expansion. BUB1 staining serves as a readout for efficient KNL1 depletion. Scale bar, 5 μm. (B) Immunofluorescence images showing that exogenous expression of GFP::ROD promotes kinetochore expansion in KNL1-depleted cells. As in (A), efficient KNL1 depletion was confirmed by co-staining the same cells for BUB1. Note that the expanded domains are only loosely associated with the centromere, marked by CENP-C. Scale bar, 5 μm. (C) Examples of detached crescents and rings in GFP::ROD-expressing cells depleted of KNL1, showing co-localization with other kinetochore components. Scale bar, 2 μm. (D) Immunofluorescence images showing that GFP::ROD is unable to support kinetochore expansion in the absence of SPDL1. Scale bar, 5μm. See also Figure S3.

Because depletion of ROD prevented kinetochore expansion, we next asked whether over-expression of ROD could promote kinetochore expansion. Consistent with previous reports [14, 17], RNAi-mediated depletion of KNL1 reduced the amount of endogenous RZZ recruited to kinetochores, as revealed by immunostaining for ZWILCH, and kinetochore expansion was significantly suppressed (Figure 3A). In this and the following experiments, the absence of BUB1 signal at kinetochores was used to confirm efficient depletion of KNL1 (Figure 3A). Strikingly, over-expression of exogenous GFP::ROD (but not GFP::ZWILCH) produced robustly expanded kinetochores in KNL1-depleted cells (Figures 3B and S3A). GFP::ROD-containing crescents in KNL1-depleted cells were often larger than GFP::ROD crescents in cells treated with control siRNA and occasionally connected kinetochores of adjacent chromosomes (Figure 3C). GFP::ROD-containing crescents and rings that were partially or even fully detached from sister kinetochore pairs were also prevalent in KNL1-depleted cells but were never observed in cells treated with control siRNA (Figures 3B and 3C). The protein composition of detached GFP::ROD crescents was identical to the detached crescents generated by acute CDK1 inhibition (Figure 3C; data not shown). As expected, co-depletion of KNL1 and SPDL1 suppressed kinetochore expansion in cells expressing GFP::ROD (Figure 3D). We also examined the effect of a GFP::ROD mutant missing its N-terminal β-propeller domain (Δ1-375), which we previously showed localizes to kinetochores but prevents recruitment of ZWILCH and SPDL1 [15]. GFP::ROD(Δ1-375) did not support expansion, as we pointed out previously [15], and depletion of KNL1 greatly reduced the kinetochore signal of the ROD mutant compared to cells treated with control siRNA (Figures S3B and S3C).

Taken together, these experiments suggest that increased ROD levels facilitate robust kinetochore expansion, even when kinetochores have a reduced capacity for RZZ recruitment, and that this depends on SPDL1 and the ROD β-propeller. This is consistent with an expansion mechanism based on self-assembly of RZZ, for which we present direct evidence below. The GFP::ROD(Δ1-375) signal most likely represents the RZZ pool directly recruited by KNL1-BUB1 without expansion, and the GFP::ROD signal also reflects higher order assemblies of RZZ. These experiments also indicate that KNL1-BUB1 are required to tether the expanded outer domain to the core kinetochore, consistent with our findings after acute CDK1 inhibition.

### *C. elegans* ROD-1 Is Capable of Self-Assembly into Micrometer-Scale Filaments *In Vivo*

**Figure 4:**
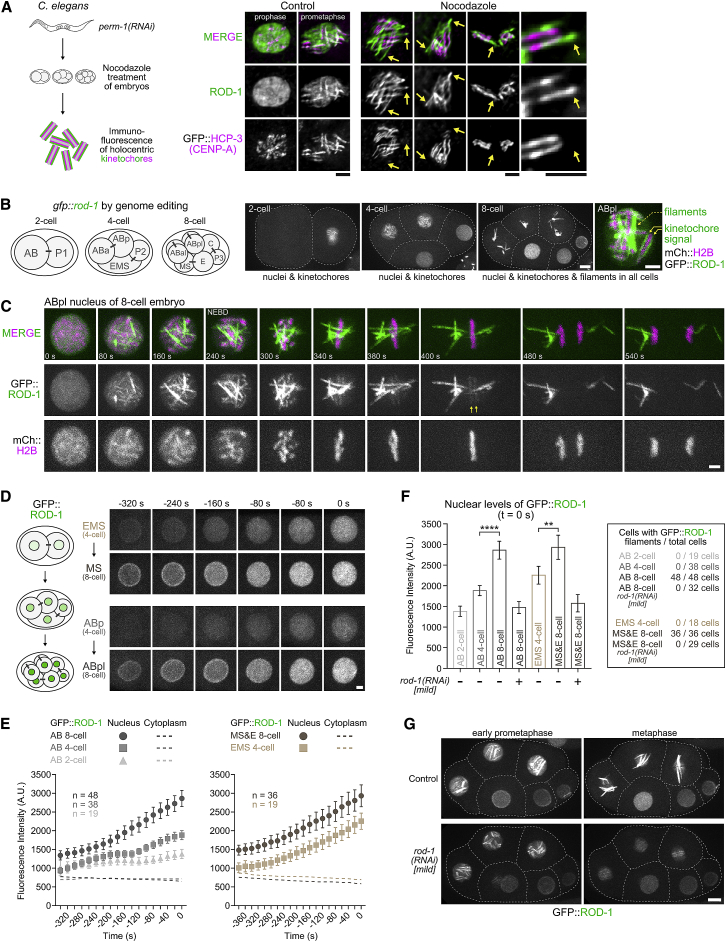
*C. elegans* ROD-1 Is Capable of Self-Assembly into Micrometer-Scale Filaments *In Vivo* (A) (Left) Schematic of experimental protocol to visualize kinetochore expansion in *C. elegans* early embryos. RNAi-mediated depletion of PERM-1 permeabilizes the eggshell of embryos, which are subsequently isolated from hermaphrodite adults, treated with nocodazole, and immunostained for ROD-1 and the centromere marker GFP::HCP-3^CENP-A^. (Right) Immunofluorescence images of mitotic embryonic cells with and without nocodazole treatment. Arrows point at filamentous kinetochore expansions containing ROD-1 that form in the absence of microtubules. Scale bars, 2 μm. (B) (Left) Schematic of the *C. elegans* early embryo at the two-, four-, and eight-cell stages. Names of individual cells are indicated. Bars connecting cells indicate they originated from the same mother cell. (Right) Selected images from a time-lapse sequence of an early embryo expressing endogenous ROD-1 tagged with GFP. GFP::ROD-1 is enriched in nuclei and localizes transiently to holocentric kinetochores in mitosis. In addition, GFP::ROD-1 starts to form filaments during mitosis at the eight-cell stage, but not earlier (see also Video S1). Dashed lines mark cell boundaries. Scale bars, 5 μm; blow-up, 2 μm. (C) Selected images from a time-lapse sequence documenting the formation of GFP::ROD-1 filaments during mitosis at the eight-cell stage (see also Video S2). mCherry::histone H2B labels chromosomes. Filaments, typically several micrometers in length, form in the nucleus before NEBD and segregate to daughter cells by clustering at spindle poles. Kinetochore-localized GFP::ROD-1 is also visible (arrows). Time point 0 refers to the last frame before the appearance of GFP::ROD-1 on filaments and kinetochores. Scale bar, 2 μm. (D) (Left) Schematic highlighting the increase in nuclear GFP::ROD-1 levels during early embryonic development. (Right) Selected images of nuclei from a time-lapse sequence of a developing embryo expressing GFP::ROD-1 that was followed from the two-cell stage to the eight-cell stage. (Top) Images show the EMS cell in the four-cell embryo, which gives rise to the MS cell in the eight-cell embryo. (Bottom) Likewise, the ABp cell gives rise to the ABpl cell. In both instances, nuclear GFP::ROD-1 levels increase gradually during the cell cycle and are significantly higher in daughter cells. Time point 0 denotes the last frame before GFP::ROD-1 appears on kinetochores (EMS and ABp) or filaments (MS and ABpl). Similar results were obtained for nuclei of the P lineage (not shown). Scale bar, 2 μm. (E) Quantification of average GFP::ROD-1 signal in nuclei and the cytoplasm in developing embryos as shown in (D). Average fluorescence intensity was determined in images acquired every 20 s, averaged for the indicated number *n* of cells from at least 8 embryos, and plotted against time. Time point 0 denotes the last frame before the appearance of GFP::ROD-1 on filaments and/or kinetochores. Values are shown as mean ± 95% confidence interval for nuclear signal and as the mean for cytoplasmic signal. (F) (Left) Quantification of nuclear GFP::ROD-1 levels in cells at different developmental stages. Measurements correspond to the last frame before GFP::ROD-1 appears on filaments and/or kinetochores (time point 0 s), showing a significant increase of nuclear signal at the eight-cell stage. Mild *rod-1(RNAi)* was used to reduce GFP::ROD-1 levels. Values are shown as mean ± 95% confidence interval. Statistical significance was determined by one-way ANOVA followed by Bonferroni's multiple comparison test. ^∗∗∗∗^p < 0.0001; ^∗∗^p < 0.01. (Right) Table showing that filament formation commences strictly at the eight-cell stage and can be completely suppressed by mildly reducing GFP::ROD-1 levels. (G) Selected images from eight-cell embryos whose AB lineage cells are going through mitosis. Mild depletion of GFP::ROD-1 slightly lowers enrichment in nuclei, which suppresses filament formation but does not prevent GFP::ROD-1 localization to kinetochores. Scale bar, 5 μm. See also Figure S4.

Rod proteins share a common architecture with membrane coat precursors, which self-assemble into higher order structures [16]. Self-assembly of ROD into filaments could underlie the central role of ROD in kinetochore expansion that we describe above, but there is currently no evidence that Rod proteins can form oligomeric assemblies. In our search for such evidence, we turned to the nematode *C. elegans*. In mature oocytes and meiosis I embryos, small filamentous structures, also referred to as “linear elements,” are present in the cytoplasm and prominently encircle the bivalent chromosomes [27, 28] (Figures S4A and S4B). Linear elements contain RZZ and other kinetochore proteins, including KNL-1, BUB-1, SPDL-1, and dynein [27, 28]. Depletion of ROD-1 prevented the formation of linear elements (Figures S4A and S4B), suggesting that they may represent oligomeric assemblies of RZZ. To ask whether RZZ is also present in filamentous structures during mitotic divisions, we immunostained early embryos with an antibody against the ROD-1 β-propeller. ROD-1 was prominently enriched in prophase nuclei and during prometaphase was present on holocentric chromosomes, where it co-localized with the centromere marker GFP::HCP-3^CENP-A^ (Figure 4A), as expected from prior work [15]. Strikingly, when embryos were incubated with nocodazole for 20 min prior to fixation, ROD-1 was detected both on kinetochores and on filamentous structures that did not co-localize with the chromosome-associated GFP::HCP-3^CENP-A^ signal (Figures 4A and S4C). Closer inspection showed that the ROD-1-containing filaments emanated from chromosomes, often appearing as an extension of the parallel holocentric kinetochore “stripes” defined by GFP::HCP-3^CENP-A^ (Figures 4A and S4C). Thus, microtubule depolymerization in *C. elegans* early embryos triggers the formation of ROD-1-containing filaments that extend beyond the core kinetochore, in analogy to kinetochore expansion in human cells. These ROD-1 filaments most likely correspond to the nocodazole-induced kinetochore “flares” documented by an earlier study [26].

Intriguingly, we found that, after tagging endogenous ROD-1 with GFP using CRISPR/Cas9-mediated genome editing (Figures S4D and S4E), GFP::ROD-1 assembled into filaments during mitosis even without nocodazole treatment (Figure 4B). Live imaging revealed that GFP::ROD-1 filaments formed with highly reproducible kinetics in mitotic nuclei at the eight-cell stage, but not during earlier divisions (Figure 4B; Video S1). In addition to forming filaments, GFP::ROD-1 localized normally to holocentric kinetochores and was enriched in nuclei (Figure 4B), just like untagged endogenous ROD-1. At the eight-cell stage, filaments formed rapidly (within ∼40 s) within nuclei about 3 min prior to nuclear envelope breakdown (NEBD), at about the same time when the GFP::ROD-1 signal first appeared on kinetochores (Figure 4C; Video S2). GFP::ROD-1 filaments remained separate from mitotic kinetochores, however, and segregated to daughter cells by clustering at spindle poles (Figures 4C and S4F; Video S2). At the end of mitosis, the filaments remained associated with centrosomes and largely disassembled before forming again in nuclei prior to NEBD of the subsequent division (Figure S4G; Video S2). Interestingly, depletion of SPDL-1 had no effect on the formation of GFP::ROD-1 filaments but prevented their clustering at spindle poles (Figure S4G), suggesting that the filaments become tethered to spindle poles via dynein. The appearance of transient GFP::ROD-1 filaments that form separately from kinetochores provides strong evidence that RZZ can reversibly self-assemble into micrometer-scale filaments *in vivo*. It is therefore probable that the mitotic RZZ filaments we observe after nocodazole treatment and the RZZ-dependent linear elements in meiosis also form via RZZ self-assembly.

Video S1. GFP::ROD-1 Localization Dynamics in the Early Embryo, Related to Figures 4 and S4 Video starts at anaphase in the 1-cell embryo and finishes at the 16-cell stage. Time lapse is 20 s and playback speed is 6 frames per second. Scale bar, 5 μm.

Video S2. Filament Formation of GFP::ROD-1 at the 8-Cell Stage, Related to Figures 4 and S4 Video shows AB cells going through mitosis in an 8-cell embryo co-expressing endogenous GFP::ROD-1 and transgene-encoded mCherry::histone H2B. Time lapse is 20 s and playback speed is 6 frames per second. Scale bar, 5 μm.

We sought to understand why GFP::ROD-1 filaments only appeared during mitosis at the eight-cell stage, but not during earlier mitotic divisions. Quantification of the GFP::ROD-1 signal revealed that nuclear levels of GFP::ROD-1 gradually increased with every embryonic division, as well as during the course of each cell cycle, reaching a peak before NEBD (Figures 4D–4F). Consequently, nuclear GFP::ROD-1 levels before NEBD in eight-cell embryos were significantly higher than nuclear levels in all previous divisions. This suggested a straightforward explanation for the invariant timing of filament formation, namely that GFP::ROD-1 must become sufficiently concentrated before oligomerization is triggered. To directly test this idea, we used a mild RNAi regime to slightly reduce total GFP::ROD-1 levels in the embryo (Figures 4F and 4G). In these RNAi conditions, nuclear GFP::ROD-1 levels in eight-cell embryos were comparable to those in control embryos at the two-cell stage (Figure 4F). Strikingly, GFP::ROD-1 no longer formed filaments in eight-cell embryos or at later developmental stages, despite the still prominent enrichment in nuclei and normal localization to mitotic kinetochores (Figures 4F and 4G). These results suggest that oligomerization can be triggered locally by concentrating ROD-1 above a critical threshold. For untagged endogenous ROD-1, reaching the threshold concentration may require accumulation at mitotic kinetochores. We speculate that the GFP tag on ROD-1’s β-propeller exacerbates ROD-1’s tendency for self-assembly, such that progressive nuclear enrichment during embryonic development eventually triggers filament formation in late prophase at the eight-cell stage independently of kinetochore targeting.

### The ROD-1 β-Propeller Suppresses Ubiquitous and Complete Oligomerization of ROD-1 into Filaments

**Figure 5:**
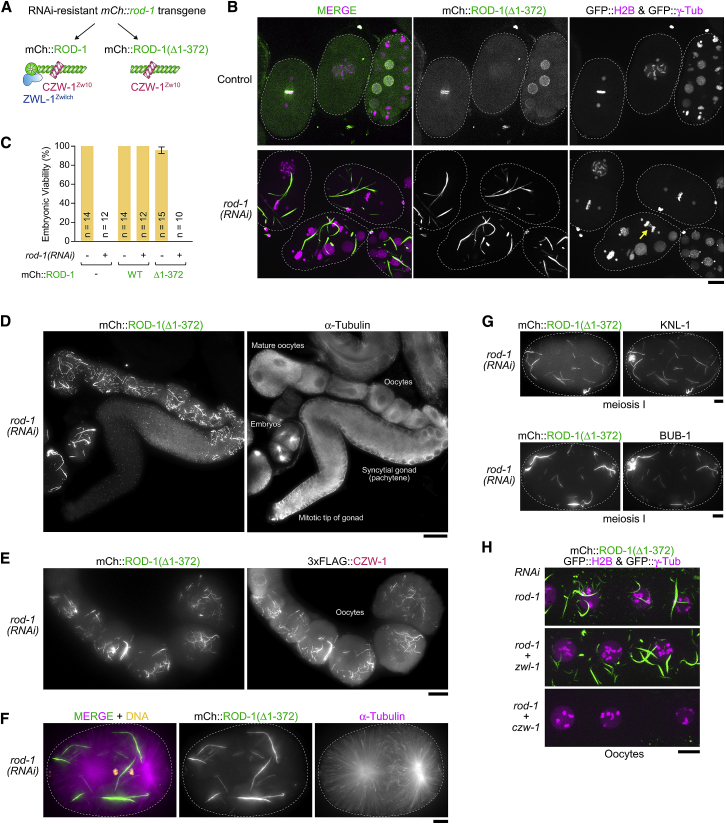
The ROD-1 β-Propeller Suppresses Ubiquitous and Complete Oligomerization of ROD-1 into Filaments (A) Schematic of the protein complexes generated by the expression of RNAi-resistant *mCherry::rod-1* transgenes integrated in single copy on chromosome II. Note that the actual RZZ complex is a dimer of the three-subunit assembly depicted here, which means mCherry::ROD-1(Δ1-372) is able to dimerize with endogenous ROD-1. (B) Selected images from time-lapse sequences in early embryos co-expressing mCherry::ROD-1(Δ1-372), GFP::histone H2B, and GFP::γ-tubulin. mCherry::ROD-1(Δ1-372) oligomerizes into filaments when endogenous ROD-1 is depleted (see also Video S3). This causes defects in chromosome segregation (arrow), because RZZ is titrated away from kinetochores. Dashed lines outline the embryos. Scale bar, 10 μm. (C) Embryonic viability assay showing that embryos expressing mCherry::ROD-1(Δ1-372) are only viable when endogenous ROD-1 is present. Values are plotted as mean ± 95% confidence interval, and *n* indicates the number of mothers whose progeny was counted. (D) Immunofluorescence image of an isolated oocyte-producing gonad, showing that mCherry::ROD-1(Δ1-372) forms filaments ubiquitously in oocytes and embryos. Scale bar, 25 μm. (E) Immunofluorescence image of maturing oocytes showing co-localization of mCherry::ROD-1(Δ1-372) with 3×FLAG-tagged CZW-1^Zw10^ on filaments. Scale bar, 10 μm. (F) Immunofluorescence image showing that mCherry::ROD-1(Δ1-372) filaments do not cluster at spindle poles during mitosis. Dashed lines outline the embryos. Scale bar, 5 μm. (G) Immunofluorescence images showing co-localization of KNL-1 and BUB-1 with mCherry::ROD-1(Δ1-372) filaments in meiosis I embryos. Dashed lines outline the embryos. Scale bars, 5 μm. (H) Fluorescence images of live oocytes showing that the formation of mCherry::ROD-1(Δ1-372) filaments depends on CZW-1^Zw10^, but not ZWL-1^Zwilch^. Scale bar, 10 μm. See also Figure S5.

In human cells, deletion of the ROD β-propeller prevents kinetochore expansion, which may at least in part be a consequence of SPDL1’s inability to bind RZZ. Because oligomerization of *C. elegans* ROD-1 into filaments was independent of SPDL-1, we wanted to address the role of the ROD-1 β-propeller in filament formation. We therefore generated animals expressing mCherry::ROD-1 with and without β-propeller (Δ1-372) from an RNAi-resistant transgene integrated in single copy at a defined chromosomal locus (Figures 5A, S5A, and S5B). Transgene expression was under the control of endogenous *rod-1* regulatory sequences, and fluorescence intensity measurements showed that mCherry::ROD-1 and mCherry::ROD-1(Δ1-372) were present at equal levels in the cytoplasm of one-cell embryos (Figure S5B). We found that mCherry::ROD-1(Δ1-372), just like full-length mCherry::ROD-1, localized to early embryonic nuclei and mitotic kinetochores when endogenous ROD-1 was present (Figure 5B). Of note, no filament formation was observed in mitotic embryonic cells for either transgene-encoded protein. By contrast, when we depleted endogenous ROD-1 by RNAi, mCherry::ROD-1(Δ1-372), but not full-length mCherry::ROD-1, oligomerized into filaments measuring up to 15 μm in length that were ubiquitously present throughout the cytoplasm of the oocyte-producing gonad and early embryo, irrespective of developmental and cell-cycle stage (Figures 5B, 5D, and S5C; Video S3). Oligomerization of mCherry::ROD-1(Δ1-372) into filaments appeared to be essentially complete judging by the lack of diffuse cytoplasmic signal (Figure 5B). Consequently, after depletion of endogenous ROD-1, mCherry::ROD-1(Δ1-372) was no longer detectable at mitotic kinetochores, and, as predicted by RZZ’s essential role at kinetochores, dividing embryos exhibited chromosome bridges in anaphase and were inviable (Figures 5B and 5C). We obtained the same results with transgene-encoded 3×FLAG::ROD-1(Δ1-372) expressed from the same chromosomal locus and regulatory elements, demonstrating that ubiquitous filament formation of ROD-1(Δ1-372) after *rod-1(RNAi)* is not a consequence of the mCherry tag (Figures S5F and S5G).

Video S3. Ubiquitous Filament Formation of ROD-1(Δ1-372), Related to Figures 5 and S5 Video shows early embryos co-expressing transgene-encoded mCherry::ROD-1(Δ1-372), GFP::histone H2B, and GFP::γ-tubulin, after depletion of endogenous ROD-1 by RNAi. Time lapse is 10 s and playback speed is 6 frames per second. Scale bar, 10 μm.

We conclude that ROD-1’s propensity to oligomerize into filaments is antagonized by its N-terminal β-propeller. The behavior of *C. elegans* ROD-1(Δ1-372) was unexpected, given that human ROD(Δ1-375) prevented kinetochore expansion. The molecular basis for this difference is currently unclear but may reflect the differential role of Spindly in the formation of high-order RZZ assemblies in the two species. The ROD-1(Δ1-372) mutant illustrates that cells must have tight regulatory mechanisms in place to control Rod’s tendency to oligomerize.

Immunostaining confirmed that endogenously tagged 3×FLAG::CZW-1^Zw10^ co-localized with mCherry::ROD-1(Δ1-372) on filaments (Figure 5E), and ZWL-1^Zwilch^ and SPDL-1 were not detectable (Figure S5D; data not shown). This agrees with prior work showing that CZW-1^Zw10^ binds to ROD-1’s Sec39 domain, which is present in ROD-1(Δ1-372), and that the ROD-1 β-propeller binds to ZWL-1^Zwilch^ and SPDL-1 [15, 33]. Unlike the GFP::ROD-1 filaments (Figures 4C, S4F, and S4G), mCherry::ROD-1(Δ1-372) filaments never clustered at mitotic spindle poles and showed no evidence of directed movement (Figure 5F; Video S3), consistent with the lack of SPDL-1 on these filaments. In the -1 oocyte and meiosis I embryo, where small ROD-1-dependent filaments are naturally present, the significantly larger filaments of mCherry::ROD-1(Δ1-372) or 3×FLAG::ROD-1(Δ1-372) were prominently decorated with KNL-1 and BUB-1 (Figures 5G, S5E, and S5G). The Mis12 complex subunit KNL-3, NDC-80, and CENP-C also localized to the filaments but to a lesser extent (data not shown). The association of kinetochore proteins with ROD-1(Δ1-372) filaments was strictly limited to the -1 oocyte and meiosis I embryo (Figures S5E and S5G), implying that their recruitment is regulated by posttranslational modifications and/or as-yet-unknown meiosis-specific factors. These results demonstrate that ROD-1(Δ1-372) and CZW-1^Zw10^ retain the ability to interact with other kinetochore proteins after assembly into higher order oligomers.

### A Complex of ROD-1(Δ1-372) and CZW-1^Zw10^ Self-Assembles Efficiently into Higher Order Oligomers *In Vitro*

**Figure 6:**
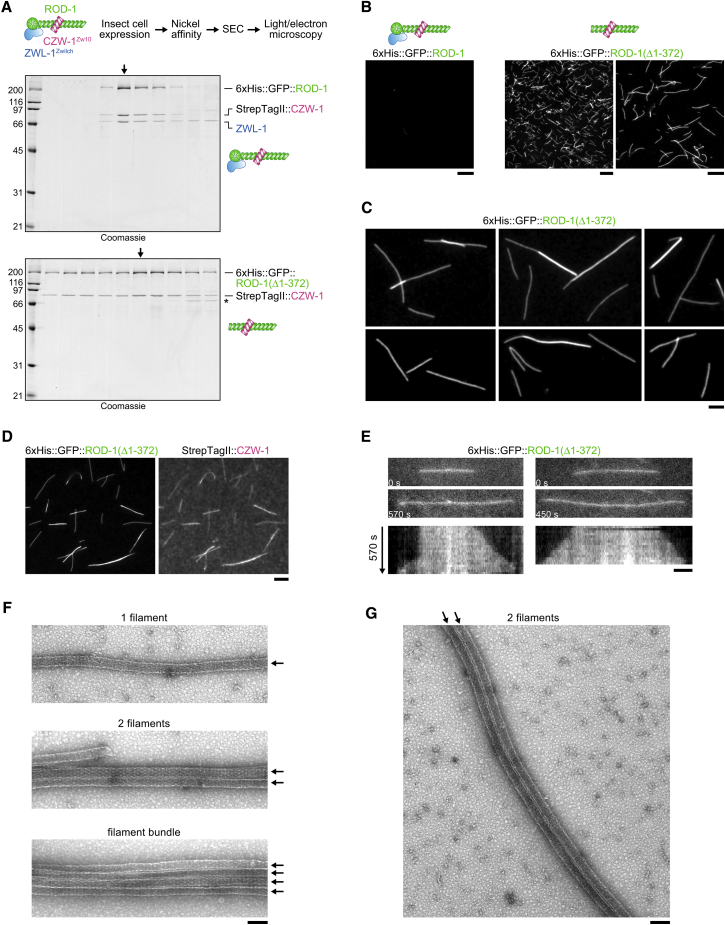
A Complex of ROD-1 without β-Propeller and CZW-1^Zw10^ Self-Assembles Efficiently into Higher Order Oligomers *In Vitro* (A) (Top) Workflow used to generate the *in vitro* data shown in this figure. (Bottom) Coomassie-stained gels showing the protein fractions after SEC. Fractions analyzed in subsequent panels are marked with an arrow. Note that purity and amount are comparable for both complexes. Molecular weight is indicated in kDa on the left. (B) Images of the protein fractions marked with an arrow in (A) examined by fluorescence light microscopy, showing that GFP::ROD-1(Δ1-372), but not full-length GFP::ROD-1, oligomerizes into micrometer-long filaments with high efficiency. Scale bars, 10 μm for left and middle image; 5 μm for right image. (C) Higher magnification views of GFP::ROD-1(Δ1-372) filaments showing evidence of lateral bundling. Note that filaments reach up to 15 μm in length. Scale bar, 2 μm. (D) Fluorescence image confirming that GFP::ROD-1(Δ1-372) co-localizes with StrepTagII::CZW-1^Zw10^ on filaments *in vitro*. Scale bar, 5 μm. (E) Selected images (top) and corresponding kymographs (bottom) from a time-lapse sequence (30 s between frames), showing that GFP::ROD-1(Δ1-372) filaments grow from both ends. In this experiment, filaments were directly examined after the nickel affinity step. Scale bar, 2 μm. (F and G) Transmission electron microscopy images of GFP::ROD-1(Δ1-372) filaments. Individual filaments (arrows) have an invariant diameter of ∼50 nm and tend to associate with each other laterally (F), often over distances of several micrometers (G). Scale bars, 100 nm. See also Figure S6.

We next sought to exploit the unique behavior of the ROD-1(Δ1-372) mutant to dissect the molecular requirements for ROD-1 oligomerization. Depletion of CZW-1^Zw10^ by RNAi completely suppressed filament formation of mCherry::ROD-1(Δ1-372) (Figure 5H), most likely because CZW-1^Zw10^ is essential for ROD-1 stability [15]. By contrast, mCherry::ROD-1(Δ1-372) filaments were not affected by depletion of ZWL-1^Zwilch^ or SPDL-1, consistent with the observation that ZWL-1^Zwilch^ and SPDL-1 did not localize to filaments. Furthermore, mCherry::ROD-1(Δ1-372) filaments readily formed after depletion of KNL-1, BUB-1, KNL-3, and NDC-80, as well as after depletion of the mitotic regulators ICP-1^INCENP^, CDK-1, and PLK-1 (data not shown). This indicated that a complex of ROD-1(Δ1-372) and CZW-1^Zw10^ may be both necessary and sufficient for efficient oligomerization into filaments. To directly test this idea, we expressed the full-length RZZ complex and the ROD-1(Δ1-372)-CZW-1^Zw10^ complex in insect Sf21 cells using baculovirus (Figure 6A). For purification and visualization, ROD-1 was N-terminally tagged with 6×His followed by GFP, and CZW-1^Zw10^ contained an N-terminal StrepTagII. After a first affinity purification step with nickel resin, size-exclusion chromatography (SEC) showed that GFP::RZZ fractionated as a complex with equal stoichiometry of its three subunits and without other obvious protein contaminants (Figure 6A). After SEC, we examined the peak fractions by fluorescence microscopy but found no evidence that GFP::ROD-1 was present in filaments (Figure 6B). We then applied the same purification procedure to the GFP::ROD-1(Δ1-372)-CZW-1^Zw10^ complex, which we obtained in similar amounts and purity as GFP::RZZ (Figure 6A). In contrast to GFP::RZZ, GFP::ROD-1(Δ1-372)-CZW-1^Zw10^ fractionated in a broader peak in SEC. Subsequent examination of peak fractions using fluorescence and transmission electron microscopy revealed that GFP::ROD-1(Δ1-372) was present in filaments that were typically several micrometers in length (up to ∼15 μm) and had an invariant width of ∼50 nm (Figures 6B–6G). Filaments frequently associated laterally with each other to form loose bundles (Figures 6C, 6F, and 6G). Staining for the StrepTagII confirmed that CZW-1^Zw10^ co-localized with GFP::ROD-1(Δ1-372) on filaments (Figure 6D). We also readily obtained the same filaments with a ROD-1(Δ1-372)-CZW-1^Zw10^ complex, confirming that the GFP tag is dispensable for oligomerization of ROD-1(Δ1-372) (Figures S6C, S6E, and S6F). If the SEC step was omitted and partially purified GFP::ROD-1(Δ1-372)-CZW-1^Zw10^ was examined directly after nickel affinity chromatography, filament formation could occasionally be followed by live imaging, which revealed that filaments grow from both ends (Figure 6E). We found that 500 mM NaCl prevented filament formation, indicating that self-assembly of ROD-1(Δ1-372)-CZW-1^Zw10^ involves electrostatic interactions (Figures S6A–S6C). After purification of ROD-1(Δ1-372)-CZW-1^Zw10^ precursors in 500 mM NaCl (Figures S6A and S6B), filaments could subsequently be generated by dialysis to 150 mM NaCl (Figures S6C–S6E). We exploited this to directly compare the propensity for filament formation of complexes containing full-length ROD-1 or ROD-1(Δ1-372), starting from an equal concentration of soluble precursors in 500 mM NaCl (Figure S6D). Under these conditions, ROD-1(Δ1-372)-containing complexes readily formed filaments after dialysis, and full-length RZZ did not, consistent with results *in vivo*.

We conclude that a purified ROD-1(Δ1-372)-CZW-1^Zw10^ complex oligomerizes efficiently into micrometer-scale filaments *in vitro* and that these filaments resemble those observed with this ROD-1 mutant *in vivo*. Thus, ROD-1(Δ1-372)-CZW-1^Zw10^ represents a minimal RZZ construct sufficient for self-assembly into higher order oligomers.

## Discussion

The RZZ complex is well established as an outer kinetochore component required for the recruitment of Mad1-Mad2 and dynein [11, 34, 35, 36, 37, 38]. Here, we present evidence that RZZ may additionally function as a structural precursor for the assembly of the dynamic outermost kinetochore domain, which expands in early prometaphase to accelerate spindle assembly [22]. Recent work has revealed that the RZZ subunit Rod is evolutionarily related to coat proteins that form higher-order assemblies on membranes [16, 29]. We demonstrate in *C. elegans* that Rod proteins are also capable of self-assembly. Although additional components most likely regulate RZZ self-assembly *in vivo*, we show *in vitro* that a complex consisting of ROD-1 without its β-propeller and CZW-1^Zw10^ is sufficient to form micrometer-scale filaments, which have an invariant diameter of ∼50 nm. Based on the human RZZ structure [16], the precursor of these filaments most likely corresponds to a dimer of the ROD-1(Δ1-372)-CZW-1^Zw10^ subassembly. Similarly, the precursor of the RZZ filaments we observe *in vivo* is most likely a dimer of the three-subunit subassembly consisting of ROD-1, CZW-1^Zw10^, and ZWL-1^Zwilch^. Deletion of the β-propeller greatly favors oligomerization of ROD-1 *in vitro* and *in vivo*. The ROD-1 β-propeller forms a tight complex with ZWL-1^Zwilch^ [15, 33], raising the possibility that the presence of ZWL-1^Zwilch^ in RZZ has an inhibitory effect on self-assembly. However, depletion of ZWL-1^Zwilch^ in animals expressing mCherry::ROD-1 does not result in the ubiquitous formation of filaments, such as those we observe with mCherry::ROD-1(Δ1-372) [15]. This argues that the inhibitory effect of the ROD-1 β-propeller on RZZ self-assembly is direct. The extent to which CZW-1^Zw10^ contributes to self-assembly is difficult to assess, because CZW-1^Zw10^ is essential for the stability of ROD-1 [15]. Future work may reveal the spatial arrangement of precursors within ROD-1(Δ1-372)-CZW-1^Zw10^ filaments and provide insight into the self-assembly mechanism.

Based on our analysis of *C. elegans* RZZ, we suggest that human ROD’s central role in kinetochore expansion is directly related to its capacity for oligomerization. We envision that filamentous RZZ assemblies form the structural scaffold of the expanded kinetochore domain and function as dynamic recruitment platforms for corona proteins, including SAC components, the motors dynein and CENP-E, and CENP-F. In contrast to the KMN network, protein-protein interactions among components in the expanded outer domain are low affinity and therefore challenging to dissect with biochemical approaches. Our finding that the expanded domain can be detached from the underlying kinetochore, either by acute CDK1 inhibition or by combining GFP::ROD over-expression with KNL1 depletion, suggests that RZZ-dependent expansion results in a distinct multi-protein assembly that is at least transiently stable without the KMN network. Consistent with recent studies [13, 17], our findings imply that kinetochores contain two distinct pools of Mad1-Mad2: one pool recruited by the KMN network through a direct interaction with Bub1 [7, 8, 9] and another pool localized to the expanded outer domain, most likely through a direct interaction with RZZ.

Given RZZ’s propensity to form oligomeric assemblies, an important question is how this behavior is spatially restricted to kinetochores. Our observations in *C. elegans* embryos expressing GFP::ROD-1 suggest that local precursor concentration is most likely a critical parameter governing oligomerization behavior. Specifically, analysis of GFP::ROD-1 filament formation suggests that the complex may be refractory to oligomerization until a threshold density of precursors is reached. In the context of kinetochores, recruitment of RZZ by Knl1-Bub1 could bring about the spatial proximity of RZZ precursors necessary to trigger oligomerization, which then proceeds autonomously. An important implication of the mechanism we propose is that self-recruitment contributes significantly to overall RZZ levels at unattached kinetochores. Thus, even low levels of Knl1-Bub1 may support kinetochore expansion, so long as a minimal “seed” of RZZ precursors can be recruited to start filament assembly. This idea is consistent with the observation that kinetochores are able to expand in GFP::ROD-expressing cells depleted of KNL1. Although Knl1-Bub1 appear to be dispensable for expansion *per se*, our results in GFP::ROD-expressing cells show that Knl1-Bub1 are required for the physical linkage between the expanded domain and the underlying kinetochore.

We also show that RZZ’s direct binding partner SPDL1 makes an important contribution to kinetochore expansion in cultured human cells. This function of SPDL1 is separable from its function as the kinetochore adaptor for dynein-dynactin [15], because DCTN1 depletion shows that dynein-dynactin are dispensable for kinetochore expansion. The importance of human SPDL1 for kinetochore expansion indicates that the requirements for self-assembly differ between human and *C. elegans* RZZ, as *C. elegans* RZZ is able to oligomerize into filaments in the absence of SPDL-1. Human SPDL1 is recruited to RZZ through the ROD β-propeller, and deletion of the ROD β-propeller suppresses kinetochore expansion [15, 16], consistent with the idea that self-assembly of human RZZ is directly regulated by SPDL1. However, the ZWILCH(E422A/D426A) mutant suggests that SPDL1 recruitment by RZZ *per se* is not sufficient for kinetochore expansion. Prior work in *C. elegans* indicated that SPDL-1, in addition to binding the ROD-1 β-propeller, also makes contact with ZWL-1^Zwilch^ [15]. The interaction between SPDL-1 and ZWL-1^Zwilch^ involves the conserved acidic residues we mutated in human ZWILCH, raising the possibility that an interaction between SPDL1 and ZWILCH may play a role in RZZ self-assembly. Mitotic kinases have been implicated in the regulation of kinetochore expansion in *Xenopus* egg extracts [21]. It is therefore likely that kinetochore-localized kinase activity acts on RZZ and/or SPDL1 to promote self-assembly of RZZ. Mps1 is a particularly attractive candidate given that a recent study identified multiple Mps1-dependent phospho-sites on both SPDL1 and the ROD β-propeller [39].

In summary, we propose that recruitment of RZZ to kinetochores by Knl1-Bub1 locally concentrates Rod to favor self-assembly. Aided by Spindly and possibly kinase activity, this generates RZZ-based filaments that engage weakly associating corona proteins to form the supramolecular assemblies visible as crescents and fibrous coronas by light and electron microscopy, respectively. Disassembly of the expanded domain after end-on microtubule attachment is most likely achieved by dynein-dependent poleward transport of corona proteins, a process that is well documented [11, 40, 41, 42, 43]. Indeed, failure to completely disassemble the corona may contribute to the partial retention of MAD1 at microtubule-attached kinetochores that is observed in SPDL1 mutants deficient in dynein recruitment [44, 45]. Thus, RZZ not only helps build the expanded kinetochore but also recruits the motor that brings about its eventual contraction.

## STAR★Methods

### Key Resources Table

**Table:** 

REAGENT or RESOURCE	SOURCE	IDENTIFIER
**Antibodies**		

Rabbit anti-ZWILCH	Andrea Musacchio, MPI of Molecular Physiology, Dortmund, Germany	N/A
Mouse anti-ZWILCH	Andrea Musacchio	N/A
Rabbit anti-ZW10	Andrea Musacchio	N/A
Rabbit anti-BUB-1	Arshad Desai, University of California San Diego, La Jolla, CA, USA	OD31
Rabbit anti-mCherry	Arshad Desai	OD78
Rabbit anti-DSN1	Arshad Desai	OD110
Rabbit anti-KNL-1	Arshad Desai	OD33
Rabbit anti-KNL1/CASC5	Arshad Desai	OD111
Rabbit anti-ROD-1	Arshad Desai	OD216
Rabbit anti-SPDL-1	Arshad Desai	OD164
Rabbit anti-SPDL1/CCDC99	Arshad Desai	OD157
Rabbit anti-SPDL1/CCDC99	Arshad Desai	OD174
Rabbit anti-ZWL-1	Arshad Desai	OD85
Sheep anti-BUB3	Stephen Taylor, University of Manchester, UK	N/A
Sheep anti-BUBR1	Stephen Taylor	N/A
Sheep anti-CENP-F	Stephen Taylor	N/A
Goat anti-GFP	Anthony Hyman, Max Planck Institute of Molecular Cell Biology & Genetics, Dresden, Germany	N/A
Mouse anti-α-Tubulin (DM1α)	Sigma-Aldrich	Cat No.: T6199; RRID: AB_477583
Mouse anti-α-Tubulin (clone B512)	Sigma-Aldrich	Cat No.: T5168; RRID: AB_477579
Mouse anti-BUB1	Abcam	Cat No.: ab54893; RRID: AB_940664
Guinea pig anti-CENP-C	MBL	Cat No.: PD030; RRID: AB_10693556
Mouse anti-CENP-E (clone1H12)	Abcam	Cat No.: ab5093; RRID: AB_304747
Human anti-centromere antibodies (ACA)	Antibodies Incorporated	Cat No.: 15-234-0001; RRID: AB_2687472
Mouse anti-FLAG M2	Sigma-Aldrich	Cat No.: F1804; RRID: AB_262044
Mouse anti-GFP (clone 9F9.F9)	Abcam	Cat No.: ab1218; RRID: AB_298911
Mouse anti-HEC1 (clone 9G3)	Abcam	Cat No.: ab3613: RRID: AB_303949
Mouse anti-MAD1 (clone BB3-8)	Millipore	Cat No.: MABE867/6B7115
Mouse anti-MAD2	Santa Cruz Biotechnology	Cat No.: sc-65492; RRID: AB_831526
Mouse anti-nuclear pore complex antibody (clone Mab414)	Abcam	Cat No.: ab24609; RRID: AB_448181
Mouse anti-p150/DCTN1	BD Transduction Laboratories	Cat No.: 610473; RRID: AB_397845
Rabbit anti-ROD/KNTC1	[15]	GC7
Donkey anti-goat IgG, Alexa 488 conjugate	Jackson ImmunoResearch	Cat No.: 705-545-147; RRID: AB_2336933
Donkey anti-guinea pig IgG, Alexa 647 conjugate	Jackson ImmunoResearch	Cat No.: 706-605-148; RRID: AB_2340476
Donkey anti-mouse IgG, Alexa 488 conjugate	Jackson ImmunoResearch	Cat No.: 715-545-150; RRID: AB_2340846
Donkey anti-mouse IgG, Alexa 594 conjugate	Jackson ImmunoResearch	Cat No.: 715-585-150; RRID: AB_2340854
Donkey anti-mouse IgG, Alexa 647 conjugate	Jackson ImmunoResearch	Cat No.: 715-605-150; RRID: AB_2340862
Donkey anti-rabbit IgG, Alexa 488 conjugate	Jackson ImmunoResearch	Cat No.: 711-545-152; RRID: AB_2313584
Donkey anti-rabbit IgG, Alexa 594 conjugate	Jackson ImmunoResearch	Cat No.: 711-585-152; RRID: AB_2340621
Donkey anti-sheep IgG, Alexa 488 conjugate	Jackson ImmunoResearch	Cat No.: 713-545-147; RRID: AB_2340745
Bovine anti-goat IgG HRP	Jackson ImmunoResearch	Cat No.: 805-035-180; RRID: AB_2340874
Goat anti-mouse IgG HRP	Jackson ImmunoResearch	Cat No.: 115-035-003; RRID: AB_10015289
Goat anti-rabbit IgG HRP	Jackson ImmunoResearch	Cat No.: 111-035-003; RRID: AB_2313567

**Bacterial and Virus Strains**		

DH5alpha Chemically Competent *E. coli*	Thermo Fisher Scientific	Cat No.: 18265017
DH10EMBacY	Geneva Biotech	N/A
TOP10 Chemically Competent *E. coli*	Thermo Fisher Scientific	Cat No.: C404010

**Chemicals, Peptides, and Recombinant Proteins**		

RO-3306	Sigma-Aldrich	Cat No.: SML0569
Dulbecco's modified Eagle's medium	Gibco	Cat No.: 31966021
EDTA-free Complete Protease Inhibitor Cocktail	Roche	Cat No.: 11873580001
Fetal bovine serum	Gibco	Cat No.: 10500064
FuGENE 6 transfection reagent	Promega	Cat No.: E2691
GlutaMAX	Gibco	Cat No.: 35050038
HIS-Select Nickel Affinity Gel beads	Sigma-Aldrich	Cat No.: P6611-25ML
Hygromycin B	Life Technologies	Cat No.: LTI 10687-010
IgG-free BSA	Jackson ImmunoResearch	Cat No.: 001-000-161
Nocodazole	Sigma-Aldrich	Cat No.: M1404-2MG
Oligofectamine	Invitrogen	Cat No.: 12252-011
Opti-MEM	Gibco	Cat No.: 31985047
Pierce ECL Western Blotting Substrate	Thermo Fisher Scientific	Cat No.: 32106
Prolong Gold with DAPI stain	Invitrogen	Cat No.: P36930
SFM4 medium	Hyclone	Cat No.: SH30913.02
Strep-Tactin, Oyster 645 conjugate	IBA	Cat No.: 2-1553-050
X-tremeGene HP DNA Transfection Reagent	Roche	Cat No.: 000000006366244001

**Critical Commercial Assays**		

MEGAscript T3	Invitrogen	Cat No.: AM1338M
MEGAscript T7	Invitrogen	Cat No.: AM1334
NucleoSpin Gel and PCR Clean-up	Macherey-Nagel	Cat No.: 1197-2372
Nucleospin Plasmid Miniprep Kit	Macherey-Nagel	Cat No.: 1235-3358

**Experimental Models: Cell Lines**		

HeLa Flp-In T-Rex	Stephen Taylor	N/A
HeLa FRT-TO MYC::EGFP::TEV::S-peptide:: ROD/KNTC1(1-2209)	[15]	N/A
HeLa FRT-TO MYC::EGFP::TEV::S-peptide:: ROD/KNTC1(376-2209)	[15]	N/A
HeLa FRT-TO MYC::EGFP::TEV::S-peptide::ZWILCH	this study	N/A
HeLa FRT-TO MYC::EGFP::TEV::S-peptide::ZWILCH(E422A/D426A)	this study	N/A
Sf21 insect cells	Thermo Fisher Scientific	Cat No.: 11497013

**Experimental Models: Organisms/Strains**		

*C. elegans* strain N2: *wild-type* (ancestral N2 Bristol)	Caenorhabditis Genetics Center (CGC), University of Minnesota, MN, USA	WB strain: N2 (ancestral)
*C. elegans* strain GCP24: *unc-119(ed3) III; ruIs32[pAZ132; Ppie-1::GFP::his-58; unc-119(+)] III; ddIs6[Ppie-1::GFP::tbg-1; unc-119(+)] V; ltSi426[pDC192; Prod-1::mCherry::rod-1::rod-1 3’UTR; cb-unc-119(+)] II*	[15]	GCP24
*C. elegans* strain GCP287: *prtSi103[pRG499; Prod-1::mCherry::rod-1(373-2177)::rod-1 3’UTR; cb-unc-119(+)] II; unc-119(ed3) III*	this study	GCP287
*C. elegans* strain GCP303: *prtSi103[pRG499; Prod-1::mCherry::rod-1(373-2177)::rod-1 3’UTR; cb-unc-119(+)] II; unc-119(ed3) III?; ruIs32[pAZ132; Ppie-1::GFP::his-58; cb-unc-119(+)] III; ddIs6[Ppie- 1::GFP::tbg-1; cb-unc-119(+)] V*	this study	GCP303
*C. elegans* strain GCP529: *rod-1(lt62[gfp::rod-1]) IV; ltIs122[pAA64; Ppie-1::mCherry::his-58; cb-unc-119(+)]; unc-119(ed3) III*	this study	GCP529
*C. elegans* strain GCP539: *prtSi103[pRG499; Prod-1::mCherry::rod-1(373-2177)::rod-1 3’UTR; cb-unc-119(+)] II; unc-119(ed3) III; czw1[prt78(3xflag::czw-1)] IV*	this study	GCP539
*C. elegans* strain GCP736: *prtSi141[pRG972; Prod-1::3xflag::rod-1(373-2177)::rod-1 3’UTR; cb-unc-119(+)] II; unc-119(ed3)] III*	this study	GCP736
*C. elegans* strain OD347: *ltSi4[pOD833; Phcp-3::gfp::hcp-3::hcp-3 3’UTR; cb-unc-119(+)] II; hcp-3(ok1892) III*	Arshad Desai	OD347
*C. elegans* strain OD933: *ltSi426[pDC192;Prod-1::mCherry::rod-1::rod-1 3’UTR; cb-unc-119(+)] II; unc-119(ed3) III*	[15]	OD933
*C. elegans* strain OD3367: *rod-1(lt62[gfp::rod-1]) IV*	this study	OD3367

**Oligonucleotides**		

siRNA targeting ROD/KNTC1: GUAAAUAACUUGCGAGAGU	Dharmacon	Cat No.: J-006829-05-0020
siRNA targeting SPDL1/CCDC99: GAAAGGGUCUCAAACUGAA	Dharmacon	Cat No.: LU-016970-00-0005
siRNA targeting ZWILCH: GGUAAGAUGUGACAGUUCA	Dharmacon	Cat No.: J-019377-05-0005
Pool of 4 siRNAs targeting DCTN1: CUGGAGCGCUGUAUCGUAA GAAGAUCGAGAGACAGUUA GCUCAUGCCUCGUCUCAUU CGAGCUCACUACUGACUUA	Dharmacon	Cat No.: L-012874-00-0005
Pool of 4 siRNAs targeting KNL1/CASC5: GCAUGUAUCUCUUAAGGAA GAACGUGGGUACAAGAAGA CGAGUCAGCUUUGCAGAUA GCAAAUGACAGCCAGCUAA	Dharmacon	Cat No.: L-015673-00-0005
Control siRNA targeting Luciferase: CGUACGCGGAAUACUUCGA	Dharmacon	Cat No.: D-001100-01-20
Primers used for the production of dsRNA are listed in Table S1	this study	N/A

**Recombinant DNA**		

Plasmid pCFJ90: Pmyo-2::mCherry::unc-54utr	Addgene	Cat No.: 19327
Plasmid pCFJ104: Pmyo-3::mCherry::unc-54utr	Addgene	Cat No.: 19328
Plasmid pGH8: Prab-3::mCherry::unc-54utr	Addgene	Cat No.: 19359
Plasmid pCFJ601: Peft-3::Mos1 transposase	Addgene	Cat No.: 34874
Plasmid pRG387: pcDNA5/FRT/TO-MYC::EGFP::TEV::S-peptide::ZWILCH	this study	pRG387
Plasmid pRG395: pcDNA5/FRT/TO-MYC::EGFP::TEV::S-peptide::ZWILCH(E422A/D426A)	this study	pRG395
Plasmid pRG442: pACEBac1-zwl-1	[15]	pRG442
Plasmid pRG445 : pACEBac1-6xHis::TEV::rod-1(1-2177)	this study	pRG445
Plasmid pRG499: pCFJ151-Prod-1::mCherry::rod-1(373-2177)::rod-1utr	this study	pRG499
Plasmid pRG516: pACEBac1-6xHis::TEV::rod-1(373-2177)	this study	pRG516
Plasmid pRG705: pACEBac1-6xHis::TEV::GFP::rod-1(1-2177)	this study	pRG705
Plasmid pRG706: pACEBac1-6xHis::TEV::GFP::rod-1(373-2177)	this study	pRG706
Plasmid pRG733: pACEBac1-StrepTagII::czw-1	this study	pRG733
Plasmid pRG972: pCFJ151-Prod-1::3xflag::rod-1(373-2177)::rod-1utr	this study	pRG972
Plasmid pL4440_DEST for RNAi of T01H3.4 (*perm-1*) by bacterial feeding	Ahringer RNAi Collection, Source BioScience	Cat No.: DFCIp3320H0210027D
Plasmid pOG44: Flp-recombinase expression vector	Thermo Fisher Scientific	Cat No.: V600520

**Software and Algorithms**		

Andor iQ3	Andor Technology	https://andor.oxinst.com
FIJI (Image J version 2.0.0-rc-56/1.51h)	[50]	https://fiji.sc
Prism 7.0	GraphPad Software	https://www.graphpad.com
ZEN 2.3 (blue edition)	Zeiss	https://www.zeiss.com

### Contact for Reagent and Resource Sharing

Further information and requests for resources and reagents should be directed to and will be fulfilled by the Lead Contact, Reto Gassmann (rgassmann@ibmc.up.pt).

### Experimental Model and Subject Details

The wild-type *Caenorhabditis elegans* N2 strain was used to generate knock-in and transgenic strains. Animals were maintained at 20°C on standard NGM plates seeded with *Escherichia coli* OP50 bacteria. Mixed stage hermaphrodite adults were used for immunoblotting. Early embryos were used for immunofluorescence and live imaging experiments. For tissue culture experiments, a modified version of the human cervical cancer cell line HeLa, containing a single stably integrated Flp Recombination Target (FRT) site and stably expressing the tetracycline repressor protein, was used to generate stable isogenic cell lines (Flp-In T-Rex system). HeLa cells were maintained at 37°C in a 5 % CO_2_ atmosphere in Dulbecco's modified Eagle's medium (Gibco) supplemented with 10 % fetal bovine serum (Gibco), 100 units/mL penicillin, 100 units/mL streptomycin, and 2 mM GlutaMAX (Gibco).

### Method Details

#### Molecular biology

To generate constructs for recombinant protein expression in insect cells, the cDNA coding for ZWL-1, CZW-1, and ROD-1 (residues 1-2177 and 373-2177) was cloned into the pACEbac1 expression vector. ROD-1 constructs were tagged N-terminally with 6xHis::TEV or 6xHis::TEV::GFP. CZW-1 contained an N-terminal StrepTagII.

For transgene expression in HeLa cells, cDNA coding for residues 1-591 of wild-type ZWILCH and the ZWILCH mutant E422A/D426A were cloned into a pcDNA5/FRT/TO-based vector (Invitrogen) modified to contain N-terminal MYC-EGFP-TEV-S-peptide. Point mutations were subsequently introduced to make the transgene resistant to siRNA.

For endogenous GFP tagging of the *C. elegans rod-1* locus using CRISPR/Cas9-mediated genome editing, the repair template for *gfp::rod-1* included *gfp(S65C)* with introns, inserted upstream of the *rod-1* start codon via a GGRAGS linker, and the homology arms (1046 bp left; 1036 bp right). The PAM site for the guide RNA (5’-CCACAGCTTTTGCTTCGCCT-3’) was mutated from AGG to AGA in the repair template.

To generate constructs for transgenic expression of mCherry::ROD-1(373-2177) and 3xFLAG::ROD-1(373-2177) in *C. elegans*, we modified a previously used plasmid for Mos1-mediated Singly Copy Insertion (MosSCI) encoding full-length mCherry::ROD-1(1-2177) with flanking *rod-1* promoter and 3’ UTR sequences [15].

#### Genome editing and MosSCI in *C. elegans*

For CRISPR/Cas9-mediated genome editing of the *rod-1* locus, the repair template (50 ng/μL) was co-injected with two separate plasmids, one expressing guide RNA under the U6 promoter (50 ng/μL) and the other expressing Cas9 under the eft-3 promoter (30 ng/μL) into N2 worms [46, 47]. The injection mix also contained three plasmids encoding fluorescent markers [pCFJ90 (P*myo-2::mCherry*, 2.5 ng/μL), pCFJ104 (P*myo-3::mCherry*, 5 ng/μL) and pGH8 (P*rab-3::mCherry*, 10 ng/μL)] that allowed selection for F1 transgenic animals. Progeny of F1 transgenic animals were screened for integration of *gfp* by PCR using a primer within *gfp* and a primer outside the left homology arm. To generate strains stably expressing mCherry::ROD-1(373-2177) and 3xFLAG::ROD-1(373-2177), the MosSCI method was used [48]. For single-copy transgene insertion on chromosome II (locus ttTi5605), a mixture of target plasmid (10 ng/μL), transposase plasmid pCFJ601 (P*eft-3::Mos1 transposase*, 50 ng/μL), and the selection marker plasmids pGH8 (10 ng/μL), pCFJ90 (2.5 ng/μL), and pCFJ104 (5 ng/μL) was injected into strain EG6429. Insertions were confirmed by PCR and sequencing. Strains were outcrossed 6x with the wild-type N2 strain and other fluorescent markers were subsequently introduced by mating.

#### Transgenic cell lines

Stable isogenic HeLa Flp-In T-REx cell lines expressing GFP::ZWILCH and GFP::ZWILCH(E422A/D426A) were generated by FRT/Flp-mediated recombination. Plasmids containing the ZWILCH transgenes were co-transfected with pOG44, a plasmid for expression of Flp recombinase (Invitrogen), into HeLa Flp-In T-Rex cells using FuGENE 6 (Promega). Following selection in 200 μg/mL hygromycin B (Life Technologies), colonies were pooled and expanded. Insertions were verified by PCR and sequencing.

#### RNA interference and drug treatments in HeLa cells

Cells were seeded on 12-mm poly-L-lysine-coated coverslips in 12-well plates 24 hr prior to transfection with siRNAs. Cells were transfected with 100 nM siRNAs (Dharmacon On-Target plus) targeting ROD/KNTC1, DCTN1, KNL1/CASC5, ZWILCH, and SPDL1/CCDC99, using Oligofectamine (Invitrogen) and reduced-serum Opti-MEM (Gibco) according to the manufacturer’s instructions. An siRNA against luciferase was used as a control. After incubation for 6 hr, medium and fetal bovine serum (10 % final) was added to the transfection mixture. 24 hr post-transfection, the transfection mixture was replaced with fresh medium, transgene expression was induced with 0.2 μg/mL tetracycline (Sigma-Aldrich), and cells were fixed or processed for immunoblotting 20 - 24 hr later. To depolymerize microtubules prior to immunofluorescence, cells were treated with 1 μM nocodazole (Sigma-Aldrich) for 4 hr. To detach kinetochore crescents from centromeres, cells were incubated for 4 hr with 1 μM nocodazole followed by a 30-min incubation with nocodazole and 10 μM CDK1 inhibitor RO-3306 (Sigma-Aldrich).

#### RNA interference in *C. elegans*

For production of double stranded RNA (dsRNA), oligos with tails containing T3 and T7 promoters were used to amplify regions from genomic N2 DNA or cDNA. PCR reactions were cleaned (NucleoSpin Gel and PCR Clean-up, Macherey-Nagel) and used as templates for T3 and T7 transcription reactions (MEGAscript, Invitrogen). Transcription reactions were cleaned (NucleoSpin RNA Clean-up, Macherey-Nagel) and annealed in soaking buffer (3x soaking buffer is 32.7 mM Na_2_HPO_4_, 16.5 mM KH_2_PO_4_, 6.3 mM NaCl, 14.1 mM NH_4_Cl). dsRNA was delivered by injecting L4 hermaphrodites, and animals were processed for live-imaging or immunofluorescence after incubation at 20°C for 45 - 50 hr. For partial knockdown of ROD-1 in strain OD3367, adults were injected just after the onset of embryo production and incubated at 20°C for 5 hr.

#### Drug treatment in *C. elegans* embryos

The eggshell of *C. elegans* embryos was permeabilized using *perm-1(RNAi)* [49]. L4 animals were placed on a plate containing 0.005 mM IPTG and HT115 (DE3) bacteria expressing double-stranded RNA against *perm-1* and were left at 20°C for 14–18 hr. Embryos were subsequently isolated from gravid adults by dissection in a drop of 0.7x Egg Salts medium (118 mM KCl, 3.4 mM MgCl_2_, 3.4 mM CaCl_2_, 5 mM HEPES, pH 7.4) containing 50 μM nocodazole and incubated in nocodazole for 20 min before processing for immunofluorescence.

#### Embryonic viability

Embryonic viability assays were performed at 20°C. L4 hermaphrodites injected with dsRNA were grown for 40 hr on NGM plates containing OP50 bacteria, single adults were placed on new mating plates (NGM plates with a small amount of OP50 bacteria) and removed 8 hr later. The number of hatched and unhatched embryos on each plate was counted after further incubation for 16 hr.

#### Immunoblotting

Samples were resolved by 8 or 10 % SDS-PAGE and transferred to 0.2-μm nitrocellulose membranes (GE Healthcare). Membranes were blocked with 5 % non-fat dry milk in TBST (20 mM Tris, 140 mM NaCl, and 0.1 % Tween, pH 7.6) and probed overnight at 4°C with the following primary antibodies: mouse anti-α-tubulin B512, 1:5000 (Sigma-Aldrich); rabbit anti-mCherry OD78, 1 μg/μL (gift from Arshad Desai); mouse anti-FLAG M2, 1:1000 (Sigma-Aldrich); mouse anti-p150/DCTN1, 1:500 (BD Transduction Laboratories); rabbit anti-ROD/KNTC1 GC7, 1:3500 (in-house); rabbit anti-ROD-1 OD216, 1:5000 (gift from Arshad Desai); rabbit anti-SPDL1/CCDC99 OD157, 1:3000 (gift from Arshad Desai); rabbit anti-ZWILCH, 1:900 (gift from Andrea Musacchio). Membranes were washed three times with TBST, incubated with goat secondary antibodies coupled to HRP (Jackson ImmunoResearch, 1:10000) for 1 hr at room temperature, and washed again three times with TBST. Blots were visualized by chemiluminescence using Pierce ECL Western Blotting Substrate (Thermo Fisher Scientific) and x-ray film (Fujifilm). Each immunoblot was performed 2 -3 times using samples from independent experiments.

#### Indirect immunofluorescence

HeLa cells were fixed immediately after aspiration of the medium with 4 % paraformaldehyde in Phem buffer (60 mM PIPES, 25 mM HEPES, 10 mM EGTA, 2 mM MgCl_2_, pH 6.9) for 5 min at room temperature, then permeabilized for 2 min with 0.1 % Triton X-100 in Phem buffer and rinsed once with Phem buffer. For stainings with anti-ZW10 antibody, cells were fixed in methanol at -20°C for 45 min, and re-hydrated for 5 min in PBS / 0.5 % Triton X-100 followed by 5 min in PBS / 0.1 % Triton X-100. Cells were blocked for 30 min in AbDil solution (PBS, 4 % IgG-free BSA [Jackson Immuno Research], 0.1 % Triton X-100, 5 mM NaN_3_) and incubated with primary antibody over night at 4°C, diluted in AbDil (mouse anti-CENP-E clone 1H12, 1:500 [Abcam ab5093]; sheep anti-CENP-F, 1:800 [gift from Stephen Taylor]; guinea pig anti-CENP-C, 1:1500 [MBL PD030]; rabbit anti-SPDL1/CCDC99 OD174, 1:5000 [gift from Arshad Desai]; goat anti-GFP, 1:15000 [gift from Anthony Hyman]; mouse anti-GFP clone 9F9.F9, 1:1000 [Abcam ab3616]; mouse anti-p150/DCTN1, 1:500 [BD Transduction Laboratories 610473]; mouse anti-MAD1 clone BB3-8, 1:300 [Millipore MABE867]; mouse anti-MAD2, 1:500 [Santa Cruz Biotechnology sc-65492]; rabbit anti-ZWILCH, 1:900 [gift from Andrea Musacchio]; rabbit anti-ZW10, 1:300 [gift from Andrea Musacchio]; human anti-centromere antibodies ACA, 1:5000 [Antibodies Incorporated]; sheep anti-BUBR1, 1:1000 [gift from Stephen Taylor]; sheep anti-BUB3, 1:250 [gift from Stephen Taylor]; mouse anti-BUB1, 1:200 [Abcam ab54893]; mouse anti-HEC1 clone 9G3, 1:1000 [Abcam ab3616]; rabbit anti-KNL1 OD111, 1:2000 [gift from Arshad Desai]; rabbit anti-DSN1 OD110, 1:1500 [gift from Arshad Desai]. After washing for 3 x 5 min in PBS / 0.1 % Triton X-100, cells were incubated with secondary antibodies conjugated to fluorescent dyes (Alexa 488, 594, 647 [Jackson ImmunoResearch]). Cells were washed again for 3 x 5 min, rinsed in PBS and mounted in Prolong Gold with DAPI stain (Invitrogen).

For immunofluorescence of *C. elegans* embryos, 10 - 12 adult worms were dissected into 3 μL of M9 buffer (86 mM NaCl, 42 mM Na_2_HPO_4_, 22 mM KH_2_PO_4_, 1 mM MgSO_4_) on a poly-L-lysine-coated slide. A 13 mm^2^ round coverslip was placed on the 3 μL drop, and slides were plunged into liquid nitrogen. After rapid removal of the coverslip ("freeze-cracking"), embryos were fixed in -20°C methanol for 20 min. Embryos were re-hydrated for 2 x 5 min in PBS (137 mM NaCl, 2.7 mM KCl, 8.1 mM Na_2_HPO_4_, and 1.47 mM KH_2_PO_4_), blocked with AbDil (PBS with 2 % BSA, 0.1 % Triton X-100) in a humid chamber at room temperature for 30 min, and incubated with the following primary antibodies: goat anti-GFP, 1:15000; rabbit anti-mCherry OD78, 1 μg/mL; mouse anti-α-Tubulin DM1a, 1:1000 (Sigma-Aldrich); mouse anti-FLAG M2, 1:1000 (Sigma-Aldrich); rabbit anti-KNL-1 OD33, 1 μg/mL; rabbit anti-BUB-1 OD31, 1 μg/mL; rabbit anti-ZWL-1 OD85, 1 μg/mL; rabbit anti-SPDL-1 OD164, 1:7000. Antibodies OD31, OD33, OD78, OD85, and OD164 were a gift from Arshad Desai.

Images were recorded on a Zeiss Axio Observer microscope controlled by ZEN 2.3 software, using a 100x NA 1.46 Plan-Apochromat objective, an Orca Flash 4.0 camera (Hamamatsu), and an HXP 200C Illuminator (Zeiss). Images of HeLa cells were processed using the deconvolution module for ZEN 2.3. Images shown in figures correspond to maximum intensity projections of z-stacks unless otherwise indicated and are representative of at least 3 independent experiments.

#### Live-imaging of *C. elegans* embryos

Adult gravid hermaphrodite worms were dissected in a watch glass filled with Egg Salts medium (118 mM KCl, 3.4 mM MgCl_2_, 3.4 mM CaCl_2_, 5 mM HEPES, pH 7.4), and embryos were mounted on a fresh 2 % agarose pad and covered with an 18 mm x 18 mm coverslip (No. 1.5H, Marienfeld). Imaging was performed in a temperature-controlled room at 20°C using a Nikon Eclipse Ti microscope coupled to an Andor Revolution XD spinning disk confocal system, composed of an iXon Ultra 897 CCD camera (Andor Technology), a solid-state laser combiner (ALC-UVP 350i, Andor Technology), and a CSU-X1 confocal scanner (Yokogawa Electric Corporation), controlled by iQ3 software (Andor Technology). A 12 x 1 μm z-stack was acquired every 20 s using a 60x NA 1.4 or 100x NA 1.45 Plan-Apochromat objective.

#### Imaging of purified 6xHis::GFP::ROD-1(Δ1-372)-StrepTagII::CZW-1

Fractions containing purified protein complex were analyzed by putting a drop of 4 μL on an acid washed 13-mm diameter coverslip and inverting the coverslip onto a slide. For visualization of StrepTagII::CZW-1, protein fractions were first incubated for 10 min with Strep-Tactin conjugated to Oyster 645 (1:300, IBA). Images were recorded on a Zeiss Axio Observer microscope (system as described for indirect immunofluorescence) using a 40x NA 1.3 or 63x NA 1.4 Plan-Apochromat objective. For time-lapse imaging, a 5 x 1 μm z-stack was recorded every 30 s.

#### Transmission electron microscopy

Protein fractions were allowed to settle on 300 mesh Formvar-carbon-coated nickel grids and were negatively stained with a 2 % aqueous solution of uranyl acetate. Grids were examined with a JEM1400 transmission electron microscope (JEOL) operating at 120 kV. Images were acquired using a post-column high-resolution (11 megapixels) high-speed camera (SC1000 Orius, Gatan). Images shown are representative of at least 3 independent preparations imaged on different days.

#### Image analysis

Image analysis was performed using Fiji software (Image J version 2.0.0-rc-56/1.51h) [50].

##### Quantification of kinetochore volumes and fluorescence intensity in HeLa cells

Volume and intensity of kinetochore fluorescence for CENP-E, CENP-F, SPDL1, and GFP were measured using the 3D Objects Counter tool. To each image stack (0.2-μm z-steps) encompassing one cell, the Subtract Background function was applied (rolling ball radius of 5 pixels), and a mask was generated based on an empirically determined threshold that maximized the number of detected kinetochores, while minimizing the detection of false objects. The mask was re-directed to the original unprocessed image stack to determine the number of voxels and the integrated intensity for each object, which could consist of one or several closely apposed kinetochores. Object values were then summed to give the total number of voxels and the total integrated intensity for kinetochores in the image stack. To determine background intensity, the number of voxels and integrated intensity were measured for 5 separate regions in the image stack that did not contain kinetochores. Values for the 5 regions were averaged, normalized to the total number of kinetochore voxels, and subtracted from the total integrated kinetochore intensity. The total number of kinetochores in the image stack was determined separately using the CENP-C signal after maximum intensity projection of the image stack followed by thresholding, automatic particle counting, and verification by visual inspection. Finally, the total number of kinetochore voxels and the total integrated kinetochore intensity were divided by the total number of kinetochores to yield the average number of voxels and integrated intensity per kinetochore per cell. Average kinetochore volumes and integrated intensities were determined for 20 cells from 2 independent experiments.

##### Quantification of GFP::ROD-1 intensity in C. elegans embryos

Measurements of nuclear and cytoplasmic GFP::ROD-1 signal were performed after maximum intensity projection of 12 x 1 μm z-stacks, captured every 20 s in embryos developing from the 2-cell to the 8-cell stage. The mean intensity in the nucleus or in the cytoplasm of the same cell was determined, and the mean intensity of a region outside the embryos (camera background) was subtracted. The total number of embryos analyzed was derived from 4 independent experiments.

#### Bacmid and virus production for insect cell expression

For bacmid recombination [51], chemically competent DH10EmBacY cells were transformed with the expression construct of interest and incubated overnight in the shaker at 37°C in 2 mL of Luria-Bertani (LB) medium. Culture was plated on LB agar plates containing 50 μg/mL kanamycin (NZYTech), 10 μg/mL tetracycline (AppliChem), 10 μg/mL gentamycin (Sigma-Aldrich), 0.5 mM IPTG (NZYTech) and 40 μg/mL X-Gal (NZYTech), and incubated at 37°C. White colonies containing recombined bacmid were picked and cultured overnight in LB medium supplemented with kanamycin, tetracycline, and gentamycin in the shaker at 37°C. Bacmid DNA was isolated by alkaline lysis followed by isopropanol precipitation. For virus production [51], Sf21 cells in 6-well plates (10^6^ cells/well) were transfected with bacmid DNA using X-tremeGene HP DNA Transfection Reagent (Roche). 50 hr after transfection, the supernatant containing initial virus V0 was collected and used to infect a 25-mL culture (SFM4 medium, Hyclone) of Sf21 cells (0.5 × 10^6^ cells/mL) to produce V1 virus. Culture density was kept at 1 × 10^6^ cells/mL (diluted with fresh medium when needed) until cell proliferation ceased. 48 hr after proliferation arrest, V1 was harvested by centrifuging the cell suspension (5 min at 800 × g) and collecting the supernatant. V1 stock was stored at 4°C in the dark.

#### Biochemistry

For preparations of RZZ and R(Δ1-372)Z complexes, 6xHis::TEV::GFP::ROD-1, ZWL-1, and StrepTagII::CZW-1 (RZZ) or 6xHis::TEV::GFP::ROD-1(Δ1-372)/6xHis::TEV::ROD-1(Δ1-372) and StrepTagII::CZW-1 [R(Δ1-372)Z] were co-expressed in 500-mL cultures of Sf21 cells (0.8 x 10^6^ cells/mL) after co-infection with corresponding viruses. Cells were harvested by centrifugation at 800 x g for 5 min. Pellets were re-suspended in lysis buffer (50 mM HEPES, 200 mM NaCl, pH 8.0) supplemented with EDTA-free complete Protease Inhibitor Cocktail (Roche), sonicated, and cleared by centrifugation at 34000 x g for 40 min. Complexes were purified by batch affinity chromatography using HIS-Select Nickel Affinity Gel beads (Sigma-Aldrich). Beads were washed with wash buffer (50 mM HEPES, 200 mM NaCl, 10 mM imidazole, pH 8.0) and eluted on a gravity column with elution buffer (50 mM HEPES, 200 mM NaCl, 250 mM imidazole, pH 8.0). Protein complexes were further purified by size exclusion chromatography (SEC) using a Superose 6 10/300 column (GE Healthcare) equilibrated with 25 mM HEPES, 150 mM NaCl, pH 7.5. Glycerol and DTT were added to fractions containing RZZ or R(Δ1-372)Z to a final concentration of 10 % (v/v) and 1 mM, respectively. Aliquots were kept at 4°C, as filaments do not tolerate freezing.

To generate R(Δ1-372)Z filaments by dialysis from soluble precursors, complexes were purified in the presence of 500 mM NaCl. After SEC, peak fractions were pooled and concentrated to about 300 μL (∼0.15 mg/mL) using centrifugal filters (Amicon Ultra). Samples were subsequently dialyzed at 4°C overnight against 3 L of 25 mM HEPES, 150 mM NaCl, pH 7.5, using D-Tube Dialyzer Midi (Millipore). Each purification and filament formation assay was performed at least 5 times starting from independently grown batches of cells.

### Quantification and Statistical Analysis

Values in figures are reported as mean ± 95 % confidence interval. Statistical analysis was performed with GraphPad Prism 7.0 software. Normal distribution of the data was evaluated using the D'Agostino-Pearson test. The type of statistical significance test (two-tailed t test or one-way ANOVA/Bonferroni's multiple comparison test) and the samples size is indicated for each dataset in the figure legends. Differences were considered significant at p values below 0.05.

## Supplemental Information

Document S1. Figures S1–S6 and Table S1

Document S2. Article plus Supplemental Information
