# Supplementary material for: Self-Assembly of the RZZ Complex into Filaments Drives Kinetochore Expansion in the Absence of Microtubule Attachment
Source: Curr Biol. 2018 Nov 5;28(21):3408–3421.e8. doi: 10.1016/j.cub.2018.08.056 (PMC6224608; doi:10.1016/j.cub.2018.08.056)
Supplement: Document S1. Figures S1–S6 and Table S1 [file mmc1.pdf]

Current Biology, Volume *28*

Supplemental Information

Self-Assembly of the RZZ Complex into Filaments Drives Kinetochore Expansion in the Absence of Microtubule Attachment

Cláudia Pereira, Rita M. Reis, José B. Gama, Ricardo Celestino, Dhanya K. Cheerambathur, Ana X. Carvalho, and Reto Gassmann

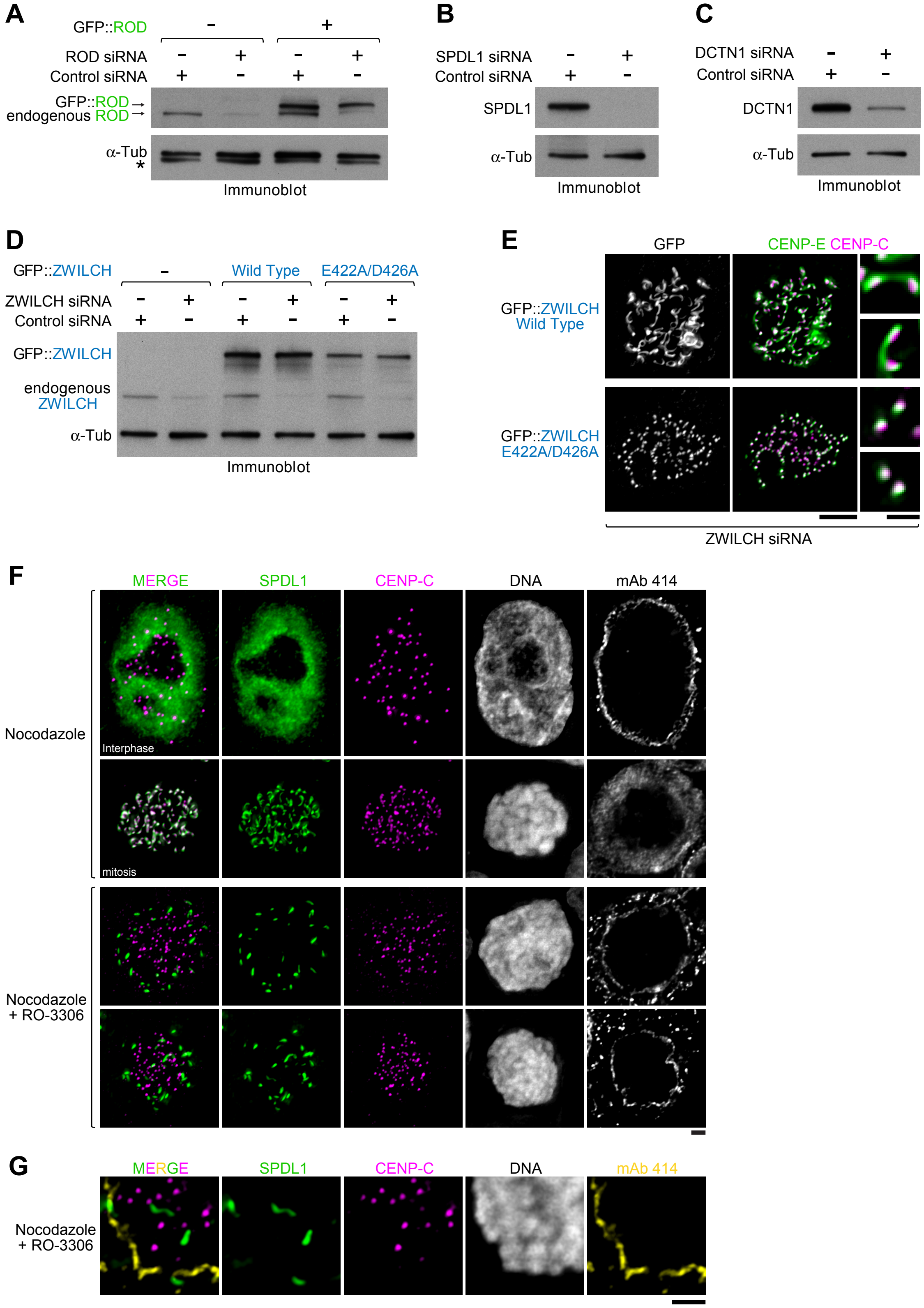

Figure S1. RNAi immunoblots and detachment of expanded kinetochore domains by acute CDK1 inhibition. Related to Figures 1 and 2.

(A) - **(D)** Immunoblots showing the efficiency of RNAi-mediated protein depletion for ROD *(A)*, SPDL1 *(B)*, DCTN1 *(C)*, and ZWILCH *(D)*, as well as expression levels of transgene-encoded GFP::ROD *(A)* and GFP::ZWILCH *(D)*. α-Tubulin was used as a loading control. A cross-reacting band in *(A)* is marked with an asterisk.

(E) Immunofluorescence images showing that the ZWILCH mutant E422A/D426A prevents the formation of CENP-E crescents. Scale bar, 5 μm; blow-ups, 1 μm.

(F) Immunofluorescence images showing the effect of acute CDK1 inhibition (10 μM RO-3306 for 30 min) on the expanded outer kinetochore, chromosome condensation, and nuclear envelope re-assembly. mAb 414 is used to detect nuclear pore complex proteins. After CDK1 inhibition, the expanded kinetochore, marked by SPDL1, detaches from the inner kinetochore, marked by CENP-C. As expected, a 30-min treatment with CDK1 inhibitor also results in partial de-condensation of chromosomes and re-assembly of the nuclear envelope.

(G) Blow up of a single z-section showing the spatial arrangement of detached kinetochore crescents relative to the inner kinetochore and the re-assembling nuclear envelope. Scale bars, 2 μm.

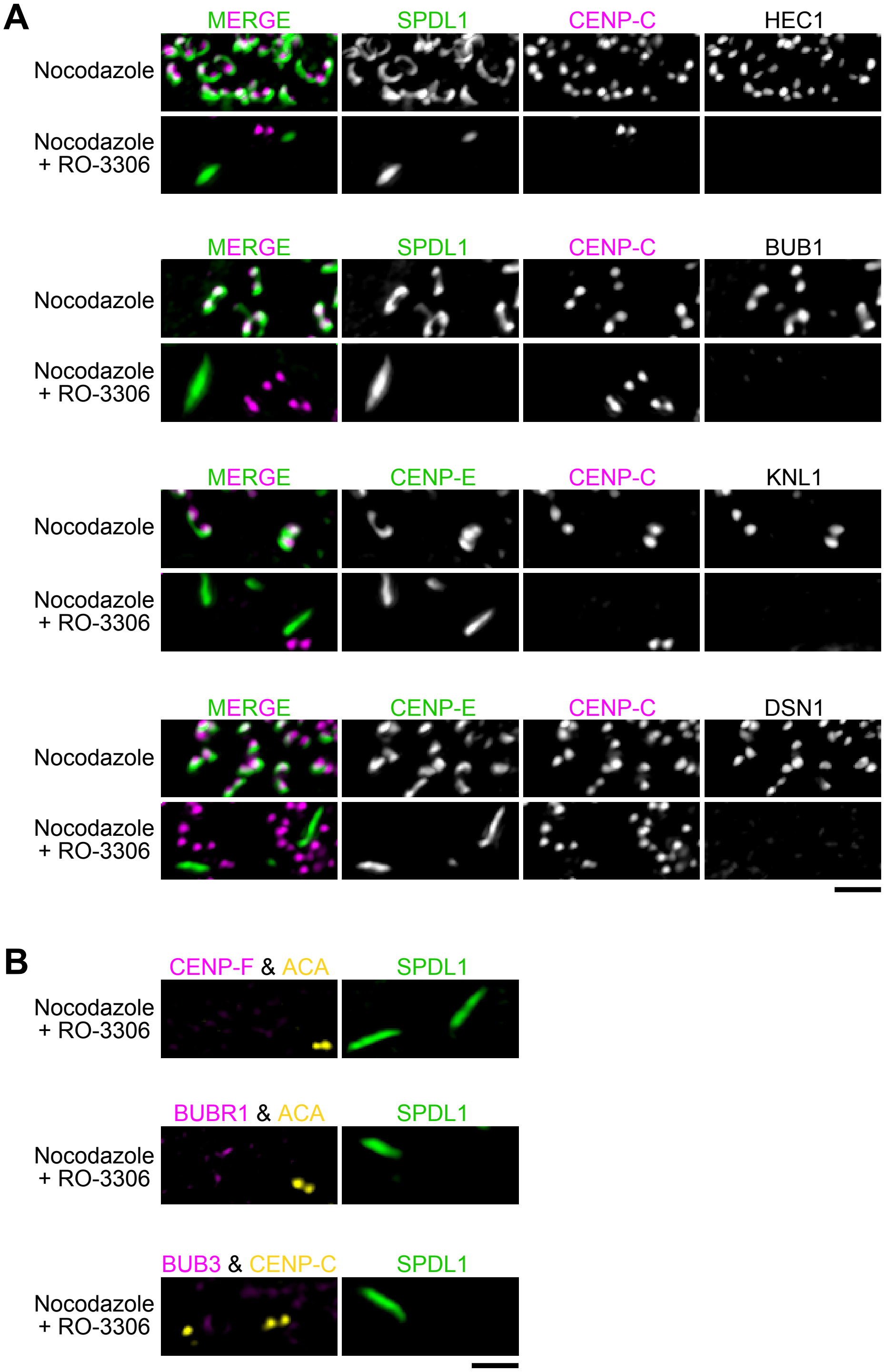

Figure S2. KMN network components do not localize to expanded kinetochore domains detached from centromeres after acute CDK1 inhibition. Related to Figure 2.

(A) Immunofluorescence images showing co-localization of HEC1, BUB1, KNL1, and DSN1 with CENP-C in nocodazole-treated cells, and the lack of signal on detached crescents, marked by CENP-E or SPDL1. Scale bar, 2 µm.

(B) Immunofluorescence images showing the absence of CENP-F, BUBR1, and BUB3 on detached crescents. Scale bar, 2 µm.

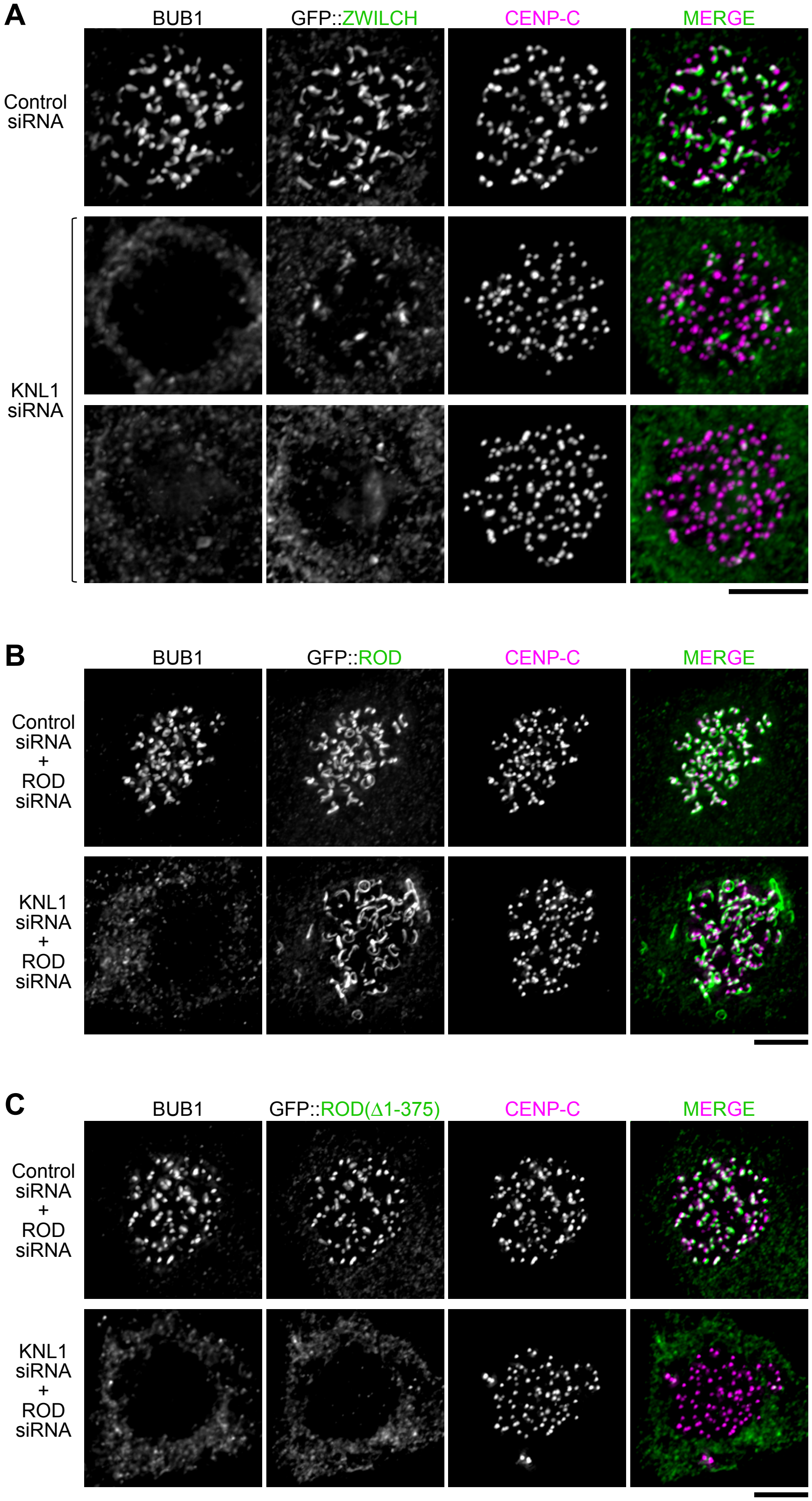

Figure S3. Kinetochore expansion after depletion of KNL1 is promoted by exogenous GFP::ROD, but not by exogenous GFP::ZWILCH, and requires the ROD β-propeller. Related to Figure 3.

(A) Immunofluorescence images showing that, unlike GFP::ROD, expression of exogenous GFP::ZWILCH does not promote kinetochore expansion after depletion of KNL1.

(B) Immunofluorescence images showing that RNAi-resistant GFP::ROD supports robust kinetochore expansion after depletion of endogenous ROD when KNL1 is co-depleted. BUB1 serves as a marker for efficient KNL1 depletion.

(C) Immunofluorescence images showing that GFP::ROD(Δ1-375) does not expand in nocodazole when endogenous ROD is depleted and cannot support expansion after co-depletion of KNL1. Scale bars, 5 µm.

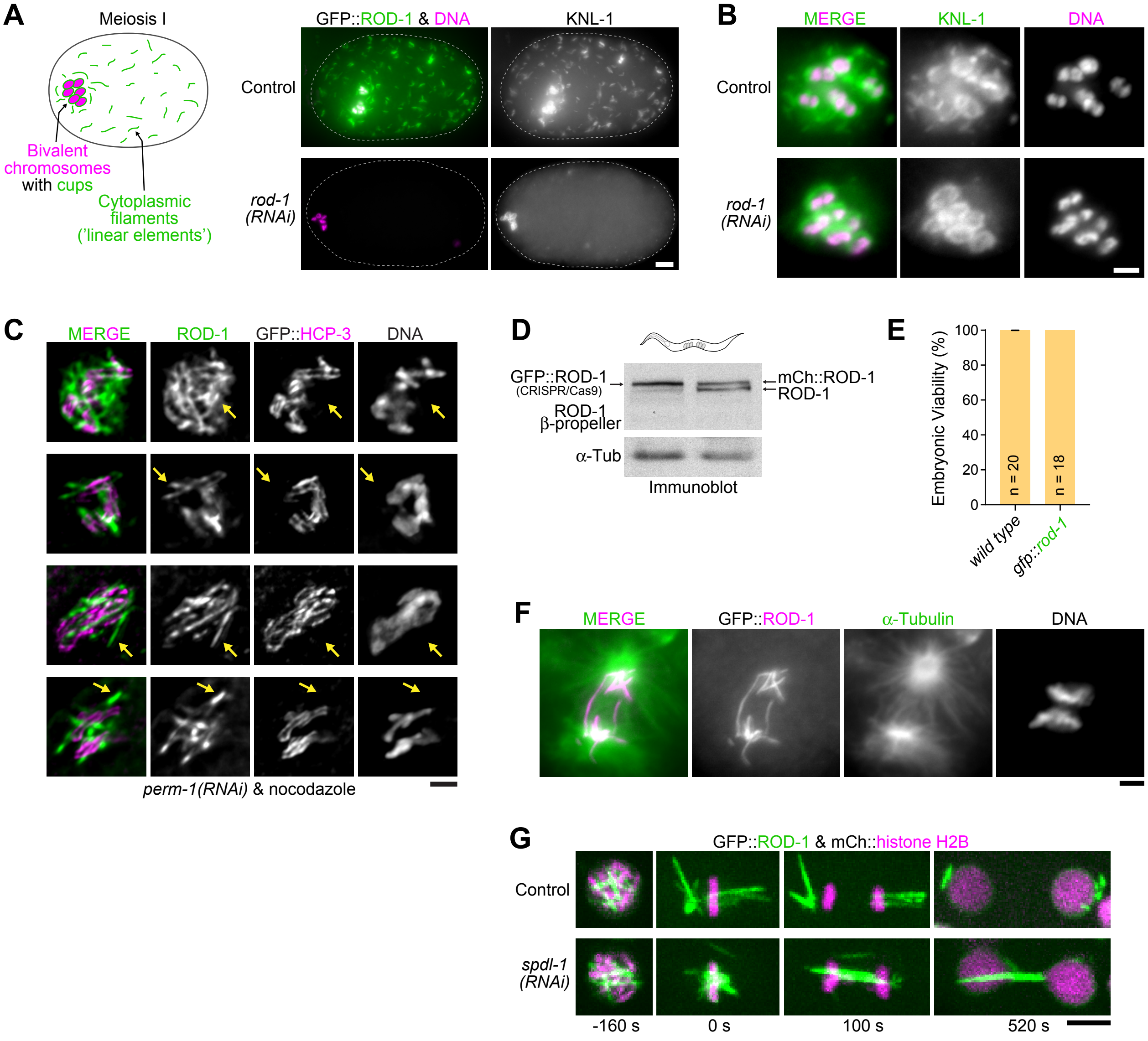

Figure S4. Characterization of ROD-1 filaments in meiosis I and the early embryo. Related to Figure 4.

(A), **(B)** Immunofluorescence images of the C. elegans meiosis I embryo, showing that GFP::ROD-1 and KNL-1 localize to small cytoplasmic filaments (linear elements) that are enriched around the bivalent chromosomes, as well as to cup-like meiotic kinetochores encircling the bivalents, as described previously [27,28]. Depletion of ROD-1 inhibits formation of linear elements but does not affect KNL-1 localization to meiotic kinetochores. Scale bars, 5 μm *(A)* and 2 μm *(B)*.

(C) Immunofluorescence images of mitotic embryonic cells after treatment with nocodazole, showing that untagged endogenous ROD-1 forms filaments (arrows) in the absence of microtubules that expand kinetochores beyond the centromeric region marked by GFP::HCP-3$^{CENP\text{-}A}$. Scale bar, 2 μm.

(D) Immunoblot of adult hermaphrodites with an antibody against the ROD-1 β-propeller, showing ROD-1 levels in animals expressing endogenous GFP::ROD-1 and in animals co-expressing endogenous untagged ROD-1 and transgene-encoded mCherry::ROD-1. α-Tubulin was used as a loading control.

(E) Tagging of endogenous ROD-1 with GFP does not affect embryonic viability. Values are plotted as mean ± 95 % confidence interval, and *n* indicates the number of mothers whose progeny was counted.

(F) Immunofluorescence image of a dividing cell in a multicellular embryo showing that GFP::ROD-1 filaments segregate to daughter cells by clustering at spindle poles. Scale bar, 2 μm.

(G) Selected images from a time-lapse sequence showing that clustering of GFP::ROD-1 filaments at spindle poles depends on SPDL-1. GFP:ROD-1 filaments that cluster at spindle poles disassemble at the end of mitosis (time point 520 s), while depletion of SPDL-1 inhibits disassembly. Scale bar, 5 μm.

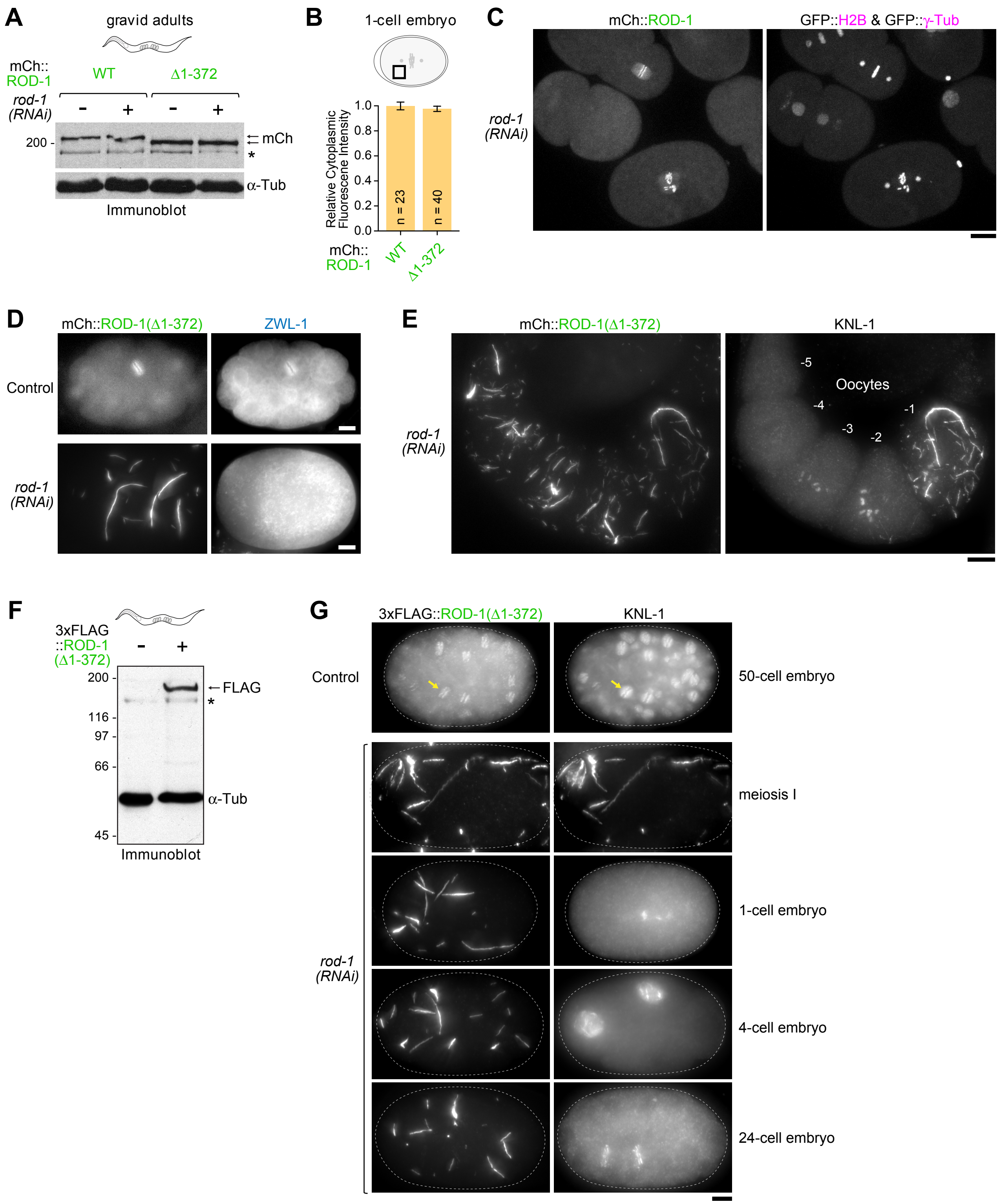

Figure S5. Characterization of cytoplasmic filaments generated by ROD-1 without its β-propeller. Related to Figure 5.
(A) Immunoblot of adult hermaphrodites with an antibody against mCherry showing levels of exogenously mCherry::ROD-1 and mCherry::ROD-1(Δ1-372). α-Tubulin was used as a loading control. A cross-reacting band is marked with an asterisk.
(B) Quantification of mCherry::ROD-1 and mCherry::ROD-1(Δ1-372) levels in the cytoplasm of 1-cell embryos, determined by measuring fluorescence intensity, as shown in the schematic. The number *n* of embryos imaged is indicated.
(C) Selected image from a time-lapse sequences showing 1- and 2-cell embryos co-expressing full-length mCherry::ROD-1, GFP::histone H2B, and GFP::α-tubulin. Depletion of endogenous ROD-1 has no effect on the localization of mCherry::ROD-1 to kinetochores, nor does it induce filament formation. Scale bar, 10 μm.
(D) Immunofluorescence image of a multi-cellular embryo *(top)* showing ZWL-1 signal at mitotic kinetochores in a control embryo and a lack of ZWL-1 signal on mCherry::ROD-1(Δ1-372) filaments in a meiotic embryo after depletion of endogenous ROD-1 *(bottom)*. Scale bars, 5 μm.
(E) Immunofluorescence image of oocytes demonstrating that KNL-1 localizes to mCherry::ROD-1(Δ1-372) filaments specifically in the most mature -1 oocyte. Scale bar, 10 μm.
(F) Immunoblot of adult hermaphrodites with an antibody against the FLAG tag, showing expression of exogenous 3xFLAG::ROD-1(Δ1-372). A cross-reacting band is marked with an asterisk. α-Tubulin was used as a loading control.
(G) Immunofluorescence images using the anti-FLAG antibody showing the localization of 3xFLAG::ROD-1(Δ1-372) in the presence (Control) and absence of endogenous ROD-1. Arrow points to kinetochore-localized 3xFLAG::ROD-1(Δ1-372). Scale bar, 5 μm.

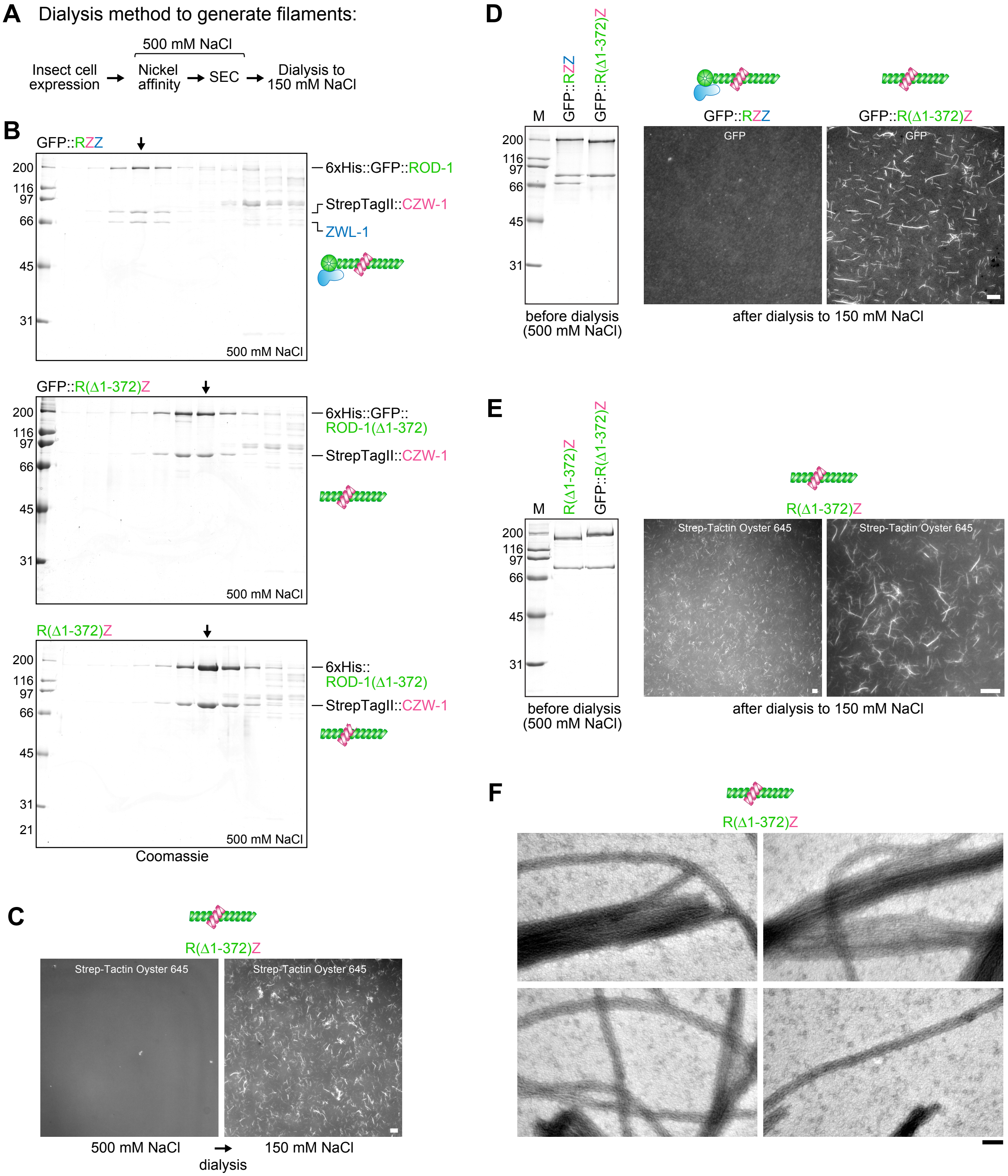

Figure S6. Dialysis from 500 mM to 150 mM NaCl induces self-assembly of ROD-1(Δ1-372)-CZW-1^{Zw10} from soluble precursors into µm-scale filaments. Related to Figure 6.

(A) Workflow used to generate filaments consisting of 6xHis::GFP::ROD-1(Δ1-372) or 6xHis::ROD-1(Δ1-372) and StrepTagII::CZW-1^{Zw10} by dialyzing purified protein fractions from 500 mM to 150 mM NaCl.

(B) Coomassie-stained gels showing protein fractions after SEC in 500 mM NaCl, which inhibits filament formation of ROD-1(Δ1-372). Fractions analyzed in subsequent panels are marked with an arrow. Molecular weight is indicated in kDa on the left.

(C) Fluorescence images showing filaments assembled with purified R(Δ1-372)Z after dialysis from 500 mM to 150 mM NaCl. Filaments were detected with Strep-Tactin conjugated to Oyster 645. Scale bar, 10 µm.

(D) *(left)* Protein gel showing full-length GFP::RZZ complex and GFP::R(Δ1-372)Z complex after purification in 500 mM NaCl. *(right)* Fluorescence images after dialysis of the samples on the left to 150 mM NaCl. Starting from identical concentrations before dialysis (1.5 mg/mL), only the GFP::R(Δ1-372)Z complex forms filaments. Scale bar, 10 µm.

(E) *(left)* Protein gel showing R(Δ1-372)Z complex with and without an N-terminal GFP tag on ROD-1 after purification in 500 mM NaCl. Sample concentration is 1.2 mg/mL for both complexes. *(right)* Fluorescence images after dialysis to 150 mM NaCl, demonstrating that R(Δ1-372)Z forms filaments as efficiently as the GFP tagged version. Filaments were detected with Strep-Tactin conjugated to Oyster 645. Scale bars, 10 µm.

(F) Transmission electron microscopy images of R(Δ1-372)Z filaments. Just like GFP::R(Δ1-372)Z filaments, filaments composed of R(Δ1-372)Z have an invariant diameter of ~50 nm and tend to form bundles. Scale bar, 100 nm.

Table S1. Oligos used for dsRNA production. Related to Figures 4 and 5.

Gene ID	Gene Name	Oligo 1 (T3 promoter)	Oligo 2 (T7 promoter)	Template
R06C7.8	*bub-1*	AATTAACCCTCACTAAAGGTGCCAAATGGAAGGACACTT	TAATACGACTCACTATAGGTCTGAGATTCTTCCGGTTCG	gDNA
F20D12.4	*czw-1*	AATTAACCCTCACTAAAGGAGTTCGATCCCGAACATGG	TAATACGACTCACTATAGGCGGAAATACTCTCGAGCACA	gDNA
Y39G10AR.13	*icp-1*	AATTAACCCTCACTAAAGGCGTCAACACCTGGACGTATG	TAATACGACTCACTATAGGGATAGGTACGTGGCGGAGTC	gDNA
C02F5.1	*knl-1*	TAATACGACTCACTATAGGCCGCTGAAATGGATACGAGT	AATTAACCCTCACTAAAGGCCATGCTAATGTCTTCACACG	gDNA
T10B5.6	*knl-3*	AATTAACCCTCACTAAAGGATGTCTCAAAAATCAAACGACACC	TAATACGACTCACTATAGGGTCGAGAAAACTTCCGTGAAG	gDNA
W01B6.9	*ndc-80*	AATTAACCCTCACTAAAGGCCCCAGTCTGAGTCAACCTC	TAATACGACTCACTATAGGCCAACTCGCTTTGAATTTCC	gDNA
F55G1.4	*rod-1*	AATTAACCCTCACTAAAGGAATGCAAATCTTTTTGGATGGG	TAATACGACTCACTATAGGCATCGACGAATTTGATTCGATC	gDNA
C06A8.5	*spdl-1*	AATTAACCCTCACTAAAGGAACGTTACCCGAATGACCAC	TAATACGACTCACTATAGGCCTAATTGAGGCATGGGTTC	cDNA
Y39G10AR.2	*zwl-1*	AATTAACCCTCACTAAAGGACTTGCGAAAACGATTTACC	TAATACGACTCACTATAGGCATTTTTGGAAGATACGAG	gDNA
